# Comparative Efficacy of Tonic Chinese Herbal Injections for Treating Sepsis or Septic Shock: A Systematic Review and Bayesian Network Meta-Analysis of Randomized Controlled Trials

**DOI:** 10.3389/fphar.2022.830030

**Published:** 2022-03-15

**Authors:** Lu Xiao, Liqing Niu, Xinyi Xu, Yuetong Zhao, Linkai Yue, Xinqiao Liu, Guiwei Li

**Affiliations:** ^1^ Department of Emergency, First Teaching Hospital of Tianjin University of Traditional Chinese Medicine, Tianjin, China; ^2^ National Clinical Research Center for Chinese Medicine Acupuncture and Moxibustion, Tianjin, China; ^3^ State Key Laboratory of Multi-Fractions Traditional Chinese Medicine, Tianjin University of Traditional Chinese Medicine, Tianjin, China

**Keywords:** network meta-analysis, sepsis, septic shock, Chinese herbal injections, combination therapy

## Abstract

**Background:** Sepsis has high mortality and is responsible for significant healthcare costs. Chinese herbal injections (CHIs) have been widely used in China as a novel and promising treatment option for sepsis. Therefore, this study assessed and ranked the effectiveness of CHIs to provide more sights for the selection of sepsis treatment.

**Method:** Eight databases were searched from their inception up to September 1, 2021. The methodological quality of included study was evaluated by the Revised Cochrane risk-of-bias tool for randomized trials. Then Bayesian network meta-analysis was performed by OpenBUGS 3.2.3 and STATA 14.0 software. The surface under the cumulative ranking curve (SUCRA) probability values were applied to rank the examined treatments. Publication bias was reflected by a funnel plot.

**Results:** A total of 50 eligible randomized controlled trials involving 3,394 participants were identified for this analysis. Five CHIs including Shenfu injection, Shenmai injection, Shengmai injection, Shenqifuzheng injection, and Huangqi injection were included. The results of the NMA and sensitivity analysis showed that Shenqifuzheng (MD = −4.48, 95% CI = −5.59 to −3.24), Shenmai (MD = −3.38, 95% CI = −4.38 to −2.39), Shenfu (MD = −2.38, 95% CI = −3.03 to −1.70) and Shengmai (MD = −1.90, 95% CI = −3.47 to −0.31) combined with Western medicine (WM) had a superior effect in improving the APACHE II score. Based on SUCRA values, Shenqifuzheng injection (95.65%) ranked highest in the APACHE II score, followed by Shenmai (74%), Shenfu (47.1%), Shengmai (35.3%) and Huangqi injection (33.2%). Among the secondary outcomes, Shenmai injection was the most favorable intervention in reducing PCT and CRP levels, and Shenqifuzheng injection was the second favorable intervention in reducing CRP level. Shenfu injection combined with WM was more effective than the other treatments in decreasing the serum IL-6 and TNF-α levels and lowering the 28-days mortality. Regarding the improvement of immune function, Shenqifuzheng injections had obvious advantages.

**Conclusion:** In conclusion, Shenqifuzheng injection was the optimum treatment regimen to improve APACHE II score, reduce CRP level, and regulate immune function. Shenfu injection was superior in reducing the expression of inflammatory factors and decreasing 28-days mortality. Nevertheless, more multicenter, diverse, and direct comparisons randomized controlled trials are needed to further confirm the results.

**Systematic Review Registration:** https://www.crd.york.ac.uk/PROSPERO/display_record.php?RecordID=254531, identifier CRD42021254531.

## Introduction

Sepsis is life-threatening organ dysfunction caused by dysregulated host response to infection (Singer et al., 2016). Sepsis and septic shock are major healthcare problem that contributes to the most causes of death in the intensive care unit (ICU) (Annane et al., 2003). Contemporary estimates indicate that more than 19 million people develop sepsis every year and that half of these will never recover; 6 million patients will die and approximately 3 million will survive with cognitive and functional impairments (Reinhart et al., 2017; Perner et al., 2018; Prescott and Angus, 2018). What’s more, the ongoing COVID-19 pandemic has infected over two million people around the world, claiming the lives of nearly 5 million people worldwide. Among the patients hospitalized with COVID-19, 26% have been treated as critical cases, which involving sepsis or even septic shock (Fan et al., 2020).

Currently, therapies for sepsis and septic shock mainly depend on fluid resuscitation, antibiotics, vasoactive agents, corticosteroid, and mechanical ventilation (Evans et al., 2021). The mainstays of treatments are early antibiotics and restoration of perfusion (IV fluids and vasopressor therapy), which are crucial for the prognosis of patients with sepsis or septic shock (Seymour et al., 2017; Kuttab et al., 2019). Timely initiation of broad-spectrum antibiotic therapy is strongly recommended in patients with sepsis and septic shock as it is associated with improved outcomes (Zhang D. et al., 2015; Seymour et al., 2017). However, the problem of antimicrobial resistance (AMR) has increased significantly worldwide (Marston et al., 2016), and decreasing the use of broad-spectrum antibiotics is a priority as this is obviously connected with the problem of AMR. This appears to be closely relevant in the ICU(De Waele et al., 2018). Additionally, optimal dosing of antibiotics in sepsis or septic shock is often not achieved with current recommended doses. The challenge is preventing underdosing while avoiding adverse effects associated with overdosing especially in those patients with acute kidney injury (AKI) due to sepsis. Moreover, fluid resuscitation is a cornerstone in the management of hemodynamic stabilization (Cheng et al., 2019; Kuttab et al., 2019). Despite being a very common therapy in the ICU, optimizing fluid administration is still challenging. Excessive fluid loading is associated with organ dysfunction and death in patients with sepsis (Sakr et al., 2017). Selection of the right kind of fluid is also a problem. Multi-center randomized controlled trials (RCTs) have shown harmful effects of synthetic colloids, notably AKI. Corticosteroids could inhibit the expression and action of many cytokines involved in the inflammatory response associated with sepsis. International guidelines recommend that IV corticosteroids are used for adults with septic shock and an ongoing requirement for vasopressor therapy (Evans et al., 2021). Although some large trials have established that corticosteroids may be effective in shock reversal and reducing ICU length of stay, it is still unclear if corticosteroids could reduce mortality of sepsis. What’s more, corticosteroids usually have some side effects, such as hyperglycemia, hypernatremia, and so on (Keh et al., 2016; Annane et al., 2018; Rochwerg et al., 2018; Venkatesh et al., 2018; Fang et al., 2019). Despite having applied all the above therapies, the mortality of sepsis remains high. Current therapies mainly rely on the timely and appropriate administration of antimicrobials and supportive therapies, but the search for pharmacotherapies modulating the host response has been unsuccessful.

In recent years, Chinese herbal injections (CHIs) especially some tonic CHIs as adjuvant treatments for sepsis or septic shock have been widely used in China. Chinese herbal medicines, i.e., Chinese herbal injections, play an essential role in the treatment of sepsis or septic shock through multicomponent, multipathway, and multitargeting abilities and have been officially recommended for the management of COVID-19 (Guo et al., 2020; Shi et al., 2021). Several Chinese treatment guidelines for sepsis management and expert consensus have been successively released for the management of sepsis (Wang and Chai, 2017; Cao et al., 2018; Zhao et al., 2019). In these guidelines and consensus, CHIs are recommended as complementary therapies based on the conventional treatment for sepsis. Shenfu and Shengmai injections are typical herbal injections officially recommended for the management of COVID-19 when patients develop into systemic inflammatory response syndrome (SIRS) and/or multiple organ dysfunction syndrome (MODS) (National Health Commission of the People’s Republic of China, 2021). Research had found that combination of Shenfu injection with standard sepsis bundle therapy significantly improved patients’ circulation, tissue perfusion, coagulation function, as well as inflammation reactions (Meiling Li et al., 2019). A meta-analysis including 17 RCTs and 860 patients with septic shock suggested that adding Shengmai injection to conventional Western medicine (WM) treatment further increased the effective rate (*p* < 0.0001) and reduced the blood lactate concentration at 12 h (*p <* 0.001), 24 h (*p* < 0.0001), and 72 h (*p =* 0.002) (Ha et al., 2019). Some studies have proved that tonic CHIs could effectively reduce the level of TNF-α, IL-6, PCT, and CRP in serum and improve the APACHE II score of patients (Pan, 2011; Qiu et al., 2012; Liu and Zhang, 2013; Cheng et al., 2018; Feng, 2019). At the same time, tonic CHIs are an effective immune-adjuvant measure for restoring monocyte immunosuppression by increasing CD4^+^ and CD4^+^/CD8^+^ levels and decreasing 28-days mortality (Zhang Z. Y. et al., 2015; Zhang et al., 2017). However, the head-to-head clinical trials comparing the efficacy of the recommended tonic CHIs are lacking up to now. Without direct evidence, it is difficult to identify the most effective one for patients with sepsis or septic shock. As a new method of evidence-based medical statistical methods, network meta-analysis (NMA) extends principles of conventional meta-analysis to the evaluation of multiple treatments in a single analysis by combining the direct and indirect evidence (Higgins and Welton, 2015; Shim et al., 2017). Another major value of NMA is that it can rank each CHI according to its effectiveness, which is important for clinicians to make the best treatment choices. Therefore, this study aimed to assess the clinical efficacy and safety of different CHIs combined with WM and provide more evidence for rational selection of CHIs for sepsis or septic shock using NMA.

## Materials and Methods

### Study Registration

This study had been prepared under the guidance of the Preferred Reporting Items for Systematic Review and Meta-Analysis (PRISMA) guidelines (in Attachment 1) (Page et al., 2021). And the study was prospectively registered on the PROSPERO platform (https://www.crd.york.ac.uk/PROSPERO/display_record.php?RecordID=254531) with an assigned registration number CRD42021254531.

### Ethics and Dissemination

All eligible studies were approved by local institutional review boards and ethical committees, and participants included were required to complete written informed consents, this study required no further ethical approval.

### Eligibility Criteria

The PICOS (participant, intervention, comparison, outcome, and study design) principle was applied in the study design.

#### Type of Included Studies

RCTs regarding CHIs for the treatment of sepsis or septic shock were included for analysis. There were no limitations on language.

#### Participants

Adults (aged 18 years or older) with sepsis or septic shock, which should be confirmed according to the diagnostic criteria (American College of Chest Physicians, 1992; Levy et al., 2003; Singer et al., 2016), patients with other critical diseases (tumor, pulmonary fibrosis, tuberculosis, and secondary respiratory failure of other systems) were excluded. No limitations existed in gender, race, or nationality.

#### Intervention

The control groups were treated with one of CHIs combined with WM, or only conventional Western medicine (WM). The experimental groups were treated with different types of CHIs combined with WM.

#### Outcomes

The primary outcome included Acute Physiology and Chronic Health Evaluation (APACHE II score). The secondary outcomes included 28-days mortality, procalcitonin (PCT), C-reactive protein (CRP), interleukin- 6 (IL-6), tumor necrosis factor-α (TNF-α), CD4^+^, CD8^+^, CD4^+^/CD8^+^, and adverse drug reactions or adverse drug events (ADRs/ADEs).

### Data Sources and Search Strategy

A comprehensive literature search was performed using the electronic databases of PubMed, the Cochrane Library, Embase, Web of Science, China National Knowledge Infrastructure (CNKI), SinoMed, Wanfang database, and the Chinese Scientific Journal database (VIP) from their inception up to September 1, 2021. The medical subject headings (MeSH) and free text words were used. Language restriction did not exist in this study. Furthermore, we manually searched the reference lists of all retrieved studies. Five different kinds of CHIs were included in this NMA: Shenfu injection, Shenmai injection, Shengmai injection, Shenqifuzheng injection, and Huangqi injection. Full details of the search strategy were shown in Attachment 2.

### Study Selection and Data Extraction

Two researchers (LXiao and LQ Niu) independently screened the studies according to the inclusion criteria. After checking for duplicate studies, the researchers eliminated reviews and irrelevant studies by reading the titles and abstracts. Then, full texts were read to select studies that met the pre-specified inclusion criteria. Inconsistencies were resolved by extensive discussion or the third researcher (GW Li). A data spreadsheet was developed with Microsoft Excel 2019 to collect relevant information. The information including eligible studies characteristics (e.g., first author, year of publication), participants characteristics (e.g., gender, age, sample), details of interventions (e.g., duration, frequency of drugs), outcomes data and factors to evaluate risk of bias were extracted and entered into the spreadsheet.

### Quality Assessment

The methodological quality of each included study was evaluated with Revised Cochrane risk-of-bias tool for randomized trials (RoB 2) (Sterne et al., 2019). The domains include the following: 1) randomisation process; 2) deviations from intended interventions; 3) missing outcome data; 4) measurement of the outcome; 5) selection of the reported result. There are some signalling questions required to answer “Yes (Y)”, “Probably Yes (PY)”, “Probably No (PN)”, “No (N)”, or “No Information (NI)” for each domain. After that, the risk of bias is categorized into three levels: high risk, some concerns, and low risk. These domain-level judgements will inform an overall risk of bias judgment for the outcome. The quality assessments were performed by two independent reviewers (LXiao and LQ Niu), and disagreements were resolved by consensus or a third opinion.

### Statistical Analysis

OpenBUGS 3.2.3 and STATA 14.0 software (Stata Corporation, College Station, TX, United States) were employed to compute calculations and prepare graphs. For binary outcomes, the combined results were calculated as odds ratios (ORs) with 95% credible intervals (CIs). For continuous outcomes, if the scales of outcomes were uniform, mean differences (MD) with 95% CIs were used, otherwise, standardized mean differences (SMD) with 95% CIs were used. When the 95% CIs of ORs did not include one and the 95% CIs of the MDs or SMDs did not contain zero, the differences between the groups were considered statistically significant. The Chi-squared test was employed to assess heterogeneity between different studies (Zheng et al., 2019). If with homogeneity (*p* ≥ 0.1, *I* ^*2*^ ≤ 50%), a fixed-effect model was adopted; If with obvious heterogeneity (*p* < 0.1, *I* ^*2*^ > 50%), a random-effect model was applied and the sources of heterogeneity were explored by sensitivity analysis. If existed closed loops, the node-splitting approach was utilized to examine the consistency between direct and indirect evidence. If the *p* > 0.05 in the node-splitting approach, it indicated that the two sources were in agreement (Dias et al., 2010).

The Markov chain Monte Carlo method was performed by using the OpenBUGS software to carry out the NMA. In OpenBUGS software, the number of iterations was set to 300,000, and the first 100,000 iterations were used for the annealing algorithm to eliminate the impact of the initial value. The network graph was constructed using STATA software to show a comparative relationship between different interventions. Surface under the cumulative ranking curve (SUCRA) probability values were applied to rank the examined treatments, and the SUCRA values of 100 and 0% were assigned to the best and worst treatments, respectively (Salanti et al., 2011; Riley et al., 2017). After that, for each treatment comparison, the comparison-adjusted funnel plot were used to assess the presence of small-study effects and publication bias if more than 10 studies were included (Salanti et al., 2014).

## Results

### Literature Selection

**FIGURE 1:**
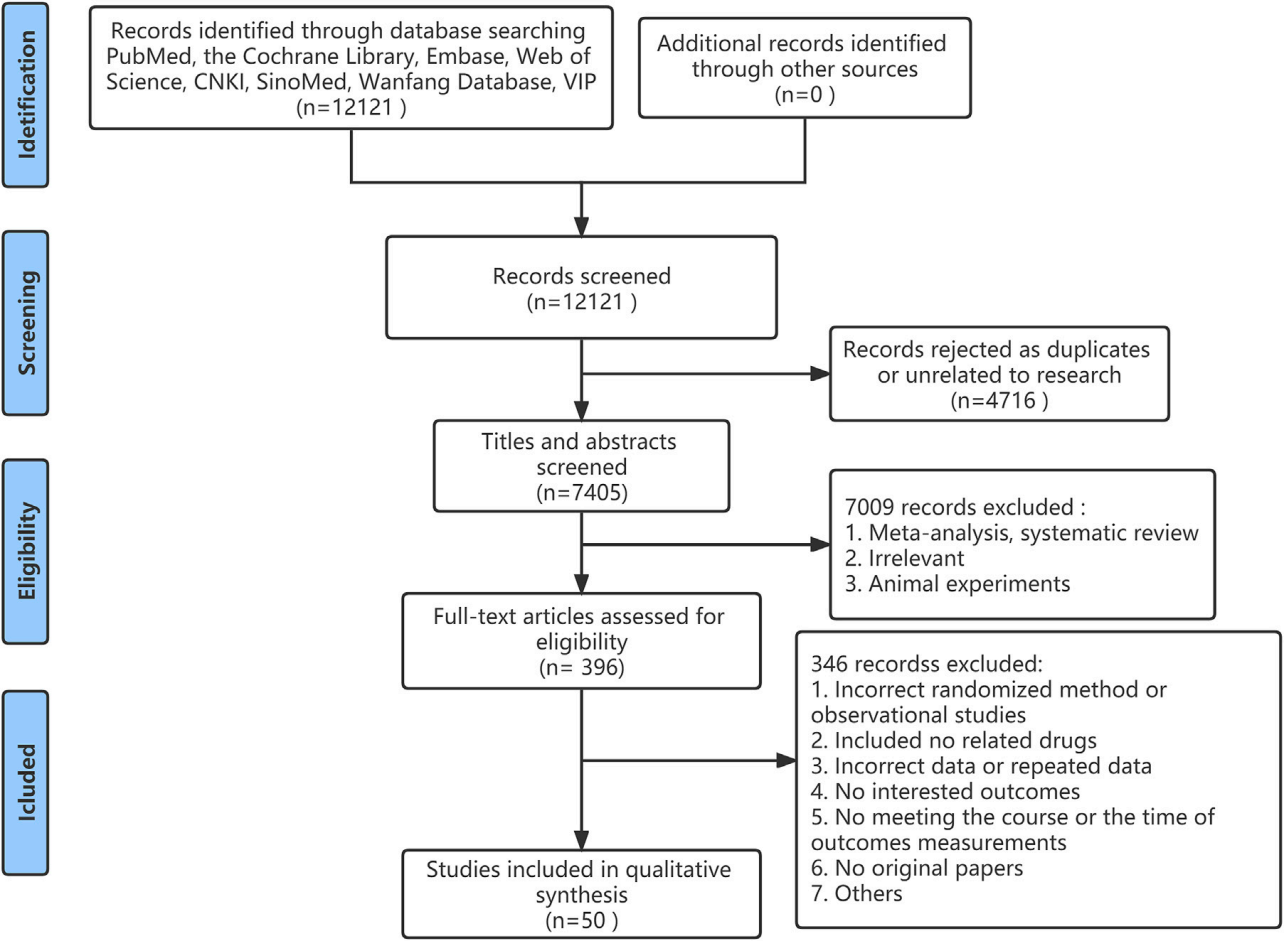
Flow diagram of study inclusion.

A total of 12,121 studies were identified from the search at first. After removing duplicates, there were 7,405 remained. By screening titles and abstracts, 7,008 studies were excluded because of reviews, irrelevant studies, and animal experiments. Afterward, 397 relevant studies were reviewed for eligibility by full-text evaluations. Finally, 50 studies that met the inclusion criteria were included in our Bayesian NMA. 347 records were excluded for the following reasons: 1) incorrect randomized method or observational studies (n = 62); 2) the use of irrelevant drugs (n = 13); 3) incorrect data or repeated data (n = 16); 4) no interested outcomes (n = 89); 5) duration of therapy or the time of outcomes measurements were not satisfied (n = 98); 6) no original papers (n = 15); (7) others (n = 54). The literature selection process was illustrated in Figure 1.

### Study Characteristics

**FIGURE 2:**
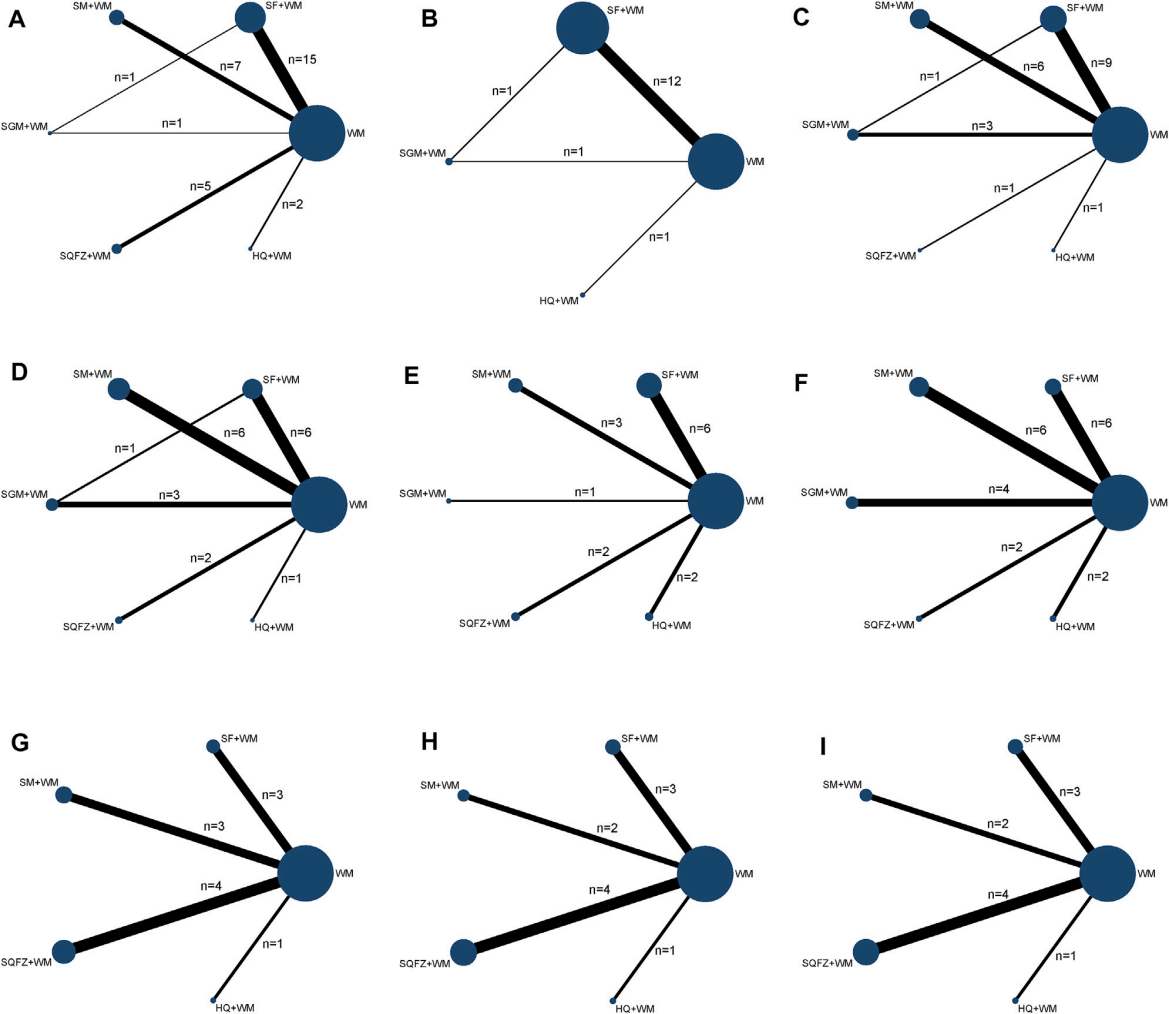
Network graph of the different outcomes. Note: **(A)**: APACHE II score; **(B)**: 28-days mortality; **(C)**: PCT; **(D)**: CRP; **(E)**: IL-6; **(F)**: TNF-α; **(G)**: CD4^+^; **(H)**: CD8^+^; **(I)**: CD4^+^/CD8^+^.

The Bayesian NMA was performed using 50 RCTs with a total of 3,394 adult patients and their sample sizes varying from 40 to 157 participants. Only one study was three-arm trial and the remaining were double-arm trials. All studies were conducted in China and published between 2008 and 2021. Five tonic CHIs were investigated including Shenfu injection (SF, n = 24), Shenmai injection (SM, n = 12), Shengmai injection (SGM, n = 5), Shenqifuzheng injection (SQFZ, n = 6), and Huangqi injection (HQ, n = 4). The control groups had been treated with conventional Western medicine of sepsis or septic shock. On the basis of the control group, the intervention of the experimental group was one of the included CHIs. The duration of treatment ranged from 7 to 14 days and the time of outcome measurements was the seventh day or eighth day after treating. The details of the study characteristics were depicted in Table 1. And the compared connections among each intervention for each outcome were displayed in Figure 2.

**TABLE 1:** Characteristics of the studies included in this meta-analysis.

Study ID	N(E/C)	Sex(M/F)	Age(years)	APACHE II score	Therapy of experiment group	Therapy of control group	Course(day)	Outcomes
Zhang et al. (2011)	36/32	38/30	E:49.3±15.5 C:50.5±17.2	E:17.58±5.77 C:18.28±5.66	Shenfu 100ml qd+WM	WM	7	②
Pan (2011)	32/22	31/23	E:54.44±3.20 C:52.05±3.59	/	Shenqifuzheng 250ml qd+WM	WM	14	①
Qiu et al. (2012)	36/32	38/30	E:49.8±10.1 C:50.3±10.18	E:17.58±5.77 C:18.28±5.66	Shenfu 100ml qd+WM	WM	7	① ④ ⑤ ⑦ ⑧ ⑨
Zheng and Pan (2014)	38/40	42/36	E:70.25±9.56 C:69.48±10.13	E:17.67±5.94 C:18.02±6.13	Shenfu 100ml qd+WM	WM	7	① ②
Ren et al. (2014)	30/30	45/15	E:66.03±15.67 C:70.43±12.46	E:19.00±6.32 C:18.07±5.93	Huangqi injection 60ml qd+WM	WM	7	① ② ⑩
Qin (2014)	34/34	40/28	E:49.1±13.6 C:50.5±16.3	/	Shengmai 60ml qd+WM	WM	7	③ ④ ⑥ ⑩
Yao (2015)	20/20	25/15	E:63.3±11.4 C:63.2±6.6	E:28.6±2.2 C:27.2±2.3	Shenfu 100ml qd+WM	WM	15	①
Zhang Z. Y. et al. (2015)	20/20	21/19	E:62.6±14.0 C:63.1±13.6	/	Shenqifuzheng 250ml qd+WM	WM	7	① ⑤ ⑥ ⑦ ⑧ ⑨
Huang (2015)	30/30	40/20	E:76.63±7.80 C:75.47±9.07	E:18.23±5.77 C:16.90±5.10	Shenfu 40ml qd+WM	WM	7	① ② ③
Liu and Yang (2018)	31/31	37/25	E:47.7±6.3 C:47.6±6.2	E:20.767±3.7 C:20.751±3.6	Shenmai 20-100ml qd+WM	WM	7	③ ④ ⑥ ⑦ ⑧
Li et al. (2021)	38/38	41/35	E:65.27±8.54 C:65.89±8.76	/	Shenfu 100ml qd+WM	WM	7	④ ⑤ ⑥ ⑩
Zhou et al. (2016)	33/32	35/30	E:63±4 C:64±3	E:20.1±1.0 C:19.1±1.2	Shenfu 120ml /+WM	WM	14	③
Wang and Wu (2016)	48/48	61/35	E:69.15±5.24 C:68.94±5.17	E:29.02±3.25 C:28.94±3.18	Shenmai 60ml q12h+WM	WM	7	① ③ ④ ⑥
Zhao et al. (2016)	35/35	37/33	E:72.6±10.3 C:74.8±12.9	/	Huangqi injection 20ml qd+WM	WM	14	⑥
Wang et al. (2016)	30/30	33/27	E:52.9±5.6 C:52.8±5.8	/	Shengmai 60ml qd+WM	WM	7	⑤ ⑥
Hu et al. (2016)	35/35	40/30	E:57.5±7.1 C:56.7±6.9	/	Shengmai 60ml qd+WM	WM	7	⑥
Zhang (2017)	36/35	39/32	E:71.43±9.21 C:69.37±10.35	E:25.78±6.89 C:25.11±7.13	Shenfu 100ml qd+WM	WM	7	①
Lu et al. (2017)	20/20	21/19	E:52.2±16.4 C:49.3±16.5	E:17.10±4.0 C:17.9±4.1	Shenmai 100ml qd+WM	WM	7	① ③ ④
Jin et al. (2017)	37/37	47/27	E:56.4±4.6 C:55.8±5.1	E:20.6±3.5 C:20.9±3.7	Shenqifuzheng 250ml qd+WM	WM	7	① ⑤ ⑥
Zhu (2017)	19/20	18/21	E:72.63±10.25 C:77.85±15.31	E:17.79±8.48 C:19.45±7.88	Shenfu 50ml Bid+WM	WM	7	① ② ③ ④ ⑦ ⑧ ⑨
Cheng et al. (2018)	34/34	44/24	E:56.65±8.17 C:57.33±7.29	E:25.77±6.83 C:26.14±5.77	Shenfu 100ml qd+WM	WM	7	① ③ ⑤ ⑥
Liu et al. (2018)	39/39	40/38	E:61.72±11.43 C:62.71±12.45	/	Shenfu 100ml Bid+WM	WM	7	③
Yan (2018)	25/25	/	E:65.51±1.62 C:65.44±1.74	/	Shenfu 100ml qd+WM	WM	7	③ ⑤ ⑥
Li (2018)	31/31	33/29	55.3±12.1	/	Shenqifuzheng 100ml qd+WM	WM	7	④ ⑦ ⑧ ⑨
Li et al. (2019)	32/32	37/27	E:49.1±15.7 C:49.2±15.4	E:17.57±5.76 C:18.27±5.65	Shenfu 100ml qd+WM	WM	7	①
Li et al. (2019)	25/25	30/20	E:67.64±14.49 C:68.84±15.80	E:25.28±7.33 C:24.68±6.19	Shenfu 60ml qd+WM	WM	7	① ② ⑤ ⑥
Pan and Chen (2020)	35/35	44/26	E:51.63±6.50 C:51.20±6.14	/	Shenfu 60ml qd+WM	WM	7	③ ④
Lei and Li (2016)	30/30	31/29	E:65.4±13.1 C:64.5±12.2	E:21.3±7.3 C:20.4±6.9	Shenfu 100ml qd+WM	WM	7	②
Zhou (2014)	30/30	33/27	E:70.15±3.45 C:69.43±2.84	E:21.17±2.92 C:20.65±2.63	Shenfu 100ml qd+WM	WM	7	① ②
Chen et al. (2015)	20/20	24/16	E:50.5±10.5 C:54.6±14.2	E:19.6±4.6 C:18.1±4.3	Shenfu 60ml qd+WM	WM	7	① ③ ④ ⑥ ⑦ ⑧ ⑨
Zhou et al. (2015)	32/32	35/29	E:53.96±10.55 C:50.32±13.74	/	Shenfu 100ml qd+WM	WM	7	② ⑤ ⑥
Cui and Dai (2016)	40/40	44/36	E:58.2±12.0 C:59.1±10.4	E:28.5±3.4 C:27.8±2.9	Shenfu 100ml q12h+WM	WM	7	① ②
Huang (2016)	20/20	24/16	E:55±6 C:57±8	E:25±5 C:26±7	Shenfu 100ml qd+WM	WM	7	②
Zhang and Zhang (2019)	67/67	72/62	E:45.3±2.5 C:49.6±2.1	E:18.73±2.54 C:19.21±2.76	Shenfu 200ml qd+WM	WM	7	①
Zhang (2019)	24/24	19/29	E:73.33±14.23 C:76.33±13.31	E:21.46±0.43 C:21.42±0.43	Shenfu 60ml qd+WM	WM	7	① ② ③ ④ ⑩
Zhang (2019)	24/25	27/22	E:73.33±14.23 C:76.28±15.85	E:21.46±0.43 C:21.84±0.42	Shengmai 40ml qd+WM	WM	7	① ② ③ ④ ⑩
Huang et al. (2010)	30/30	35/25	E:61.5±8.7 C:60.8±9.2	E:20.43±6.26 C:20.07±6.33	Shenmai 50ml q12h+WM	WM	7	① ⑤ ⑥
Ning (2011)	30/30	39/21	56.9±2.3	E:21.0±3.5 C:21.0±3.7	Shenmai 60ml qd+WM	WM	7	①
Ning XP 2012	30/30	35/25	58.2±3.6	/	Shenmai 60ml qd+WM	WM	7	⑥
Shen et al. (2014)	23/23	40/6	E:67.2±8.1 C:65.5±7.9	/	Shenmai 100ml qd+WM	WM	7	① ⑦ ⑧ ⑨
Xu et al. (2015)	40/40	44/36	60.8±9.0	/	Shenmai 10ml/h 24h+WM	WM	7	③ ④ ⑤ ⑥
Zhang (2016)	72/72	95/49	E:65.87±17.28 C:64.35±18.19	E:21.31±5.31 C:21.89±5.28	Shenmai 100ml qd+WM	WM	7	①
Zhang and Wang (2017)	41/41	47/35	E:51.32±4.57 C:50.89±5.18	/	Shenmai 60ml qd+WM	WM	7	③ ⑦ ⑨
Feng (2019)	33/33	37/29	E:62.14±18.72 C:61.78±17.33	E:21.56±2.04 C:22.53±2.42	Shenmai 10ml/h 24h+WM	WM	7	① ④ ⑤ ⑥
Chen T. F. et al. (2020)	28/34	38/24	E:73.33±14.23 C:76.28±15.94	/	Shenmai 100ml q12h+WM	WM	14	③ ④
Liu and Zhang (2013)	30/30	39/21	E:48.9±5.2 C:50.2±4.9	/	Shengmai 20ml-60ml qd+WM	WM	7	③ ④ ⑥
Ai et al. (2013)	30/30	37/23	E:55.87±13.14 C:56.53±11.17	E:20.07±4.68 C:19.47±4.75	Shenqifuzheng 250ml qd+WM	WM	7	① ③ ④ ⑦ ⑧ ⑨
Ma et al. (2018)	47/48	61/34	E:66.4±8.7 C:65.5±9.0	E:17.2±2.3 C:17.5±2.4	Shenqifuzheng 250ml qd+WM	WM	7	① ⑦ ⑧ ⑨
Ren et al. (2013)	30/30	45/15	E:66.0±15.7 C:70.4±12.5	E:19.0±6.3 C:18.1±5.9	Huangqi injection 60ml qd+WM	WM	7	③ ④ ⑤ ⑩
Chen (2008)	30/20	35/15	E:73.80±9.50 C:75.35±10.18	/	Huangqi injection 30ml q12h+WM	WM	14	① ⑤ ⑥ ⑦ ⑧ ⑨
Zhang et al. (2017)	78/79	88/69	E:59.3±16.4 C:58.6±17.2	E:18.6±6.8 C:18.3±6.5	Shenfu 100ml qd+WM	WM	7	① ②

Note: ①APACHE II score; ②28-day mortality; ③The level of PCT; ④The level of CRP; ⑤IL-6; ⑥TNF-α; ⑦CD4^+^; ⑧CD8^+^; ⑨CD4^+^/CD8^+^; ⑩ADRs/ADEs.

### Quality Assessment

**FIGURE 3:**
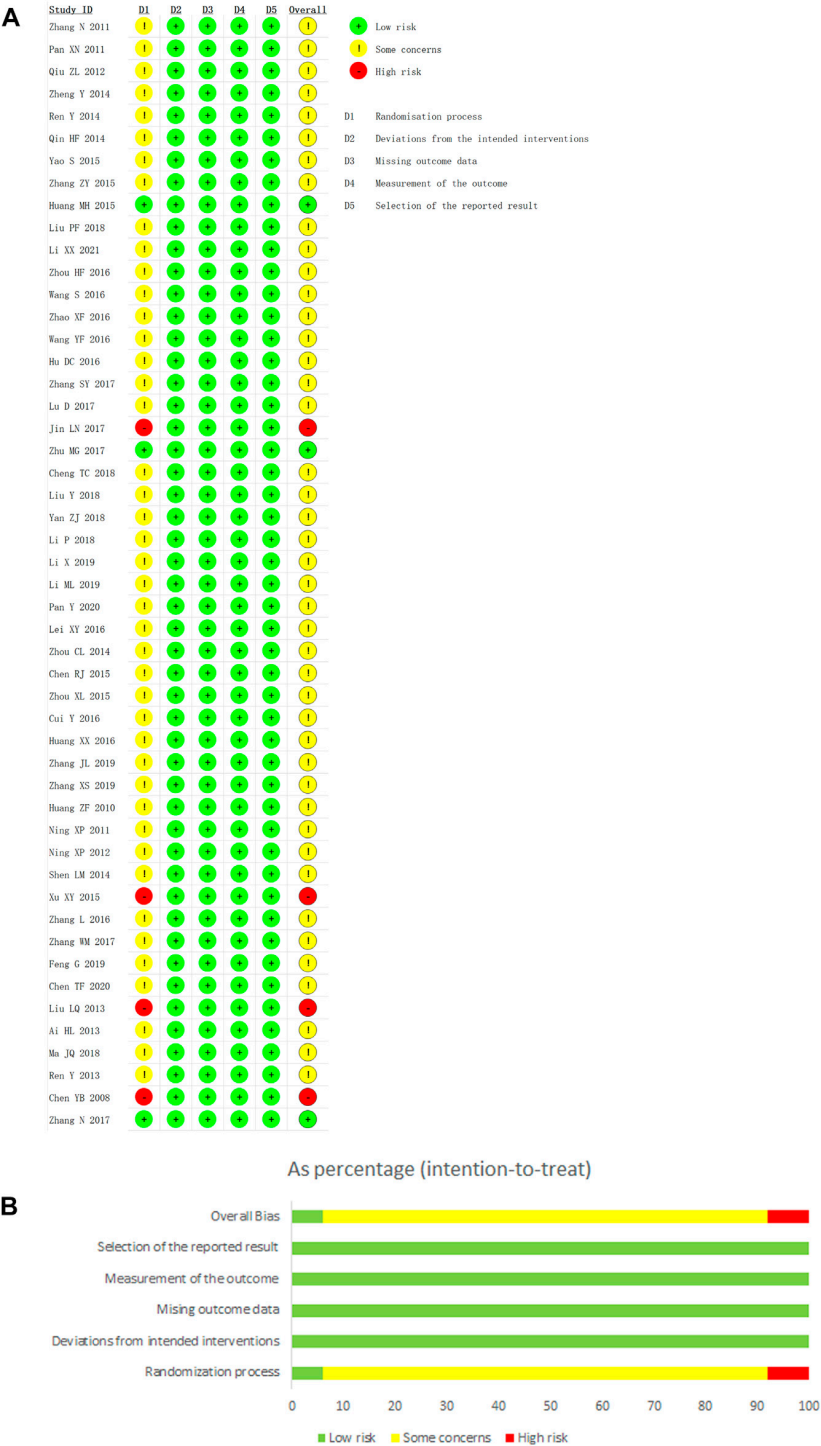
Risk of bias graph. Note: **(A)**: Risk of bias graph; **(B)**: Risk of bias summary.

We used the Revised Cochrane risk-of-bias tool for randomized studies (RoB 2) to conduct a quality evaluation. Three studies were considered as low risk for the randomization process and four studies were assessed as high risk because of their incorrect method of random sequence generation. Although the remaining 43 studies utilized correct methods of random sequence generation, their allocation concealments were not obtainable. Hence, they were considered as some concerns in randomization process. All studies were rated to have low risk of bias for deviations from intended interventions, missing outcome data, and selection of the reported result. In addition, outcomes in this study were mostly objective indicators and the methods of outcomes measurements were reasonable, so the measurements of the outcomes were assessed as low risk in all studies. In summary, four studies (8%) were considered as high risk, and 43 studies (86%) were considered as some concerns, while only three studies (6%) were considered as low risk. Further details of the risk of bias assessment were shown in Figure 3.

### Primary Outcome

#### APACHE II Score

**FIGURE 4:**
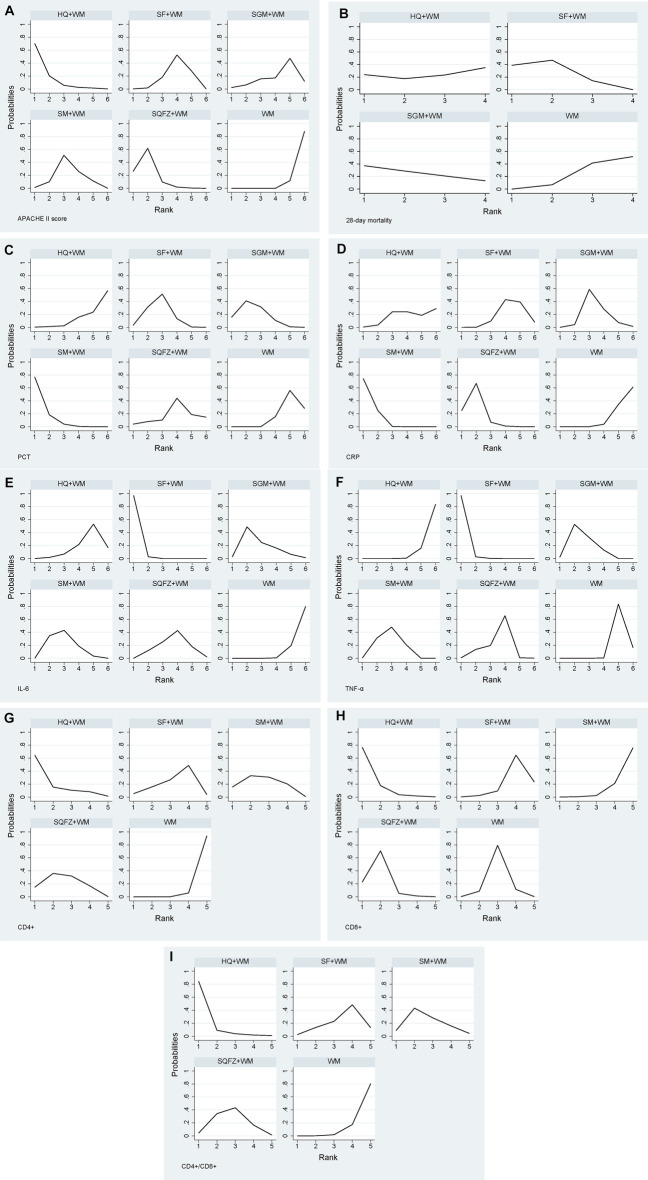
Plot of SUCRA for all different outcomes. Note: **(A)**: APACHE II score; **(B)**: 28-days mortality; **(C)**: PCT; **(D)**: CRP; **(E)**: IL-6; **(F)**: TNF-α; **(G)**: CD4^+^; **(H)**: CD8^+^; **(I)**: CD4^+^/CD8^+^.

**FIGURE 5:**
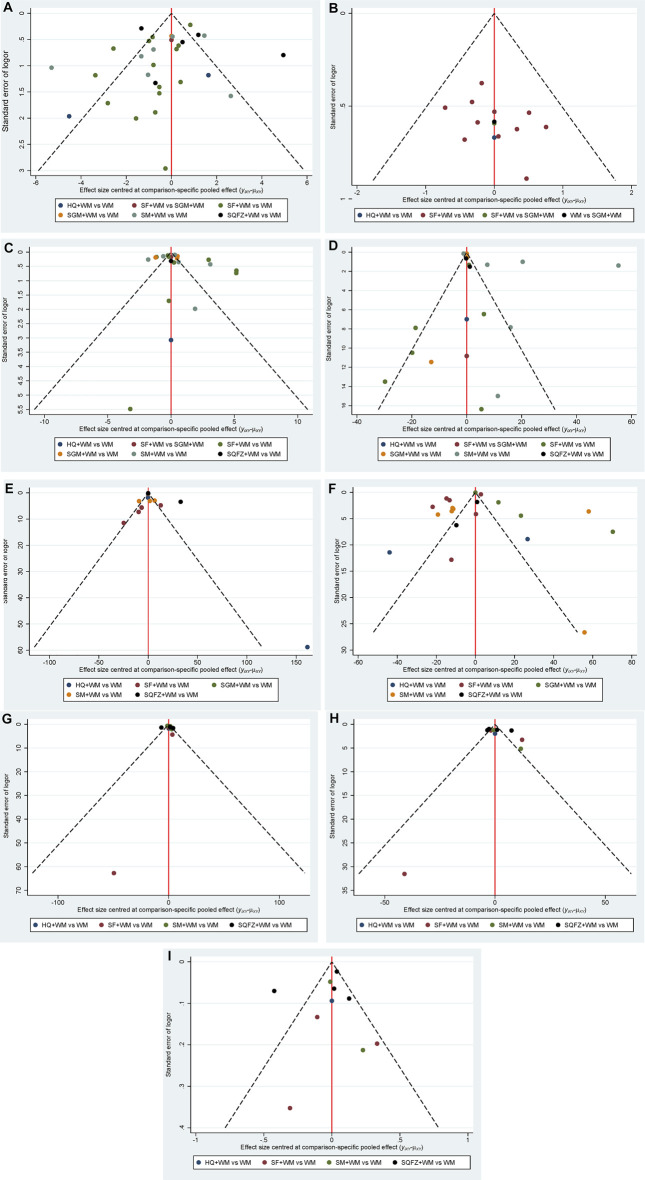
Funnel Plot. Note: **(A)**: APACHE II score; **(B)**: 28-days mortality; **(C)**: PCT; **(D)**: CRP; **(E)**: IL-6; **(F)**: TNF-α; **(G)**: CD4^+^; **(H)**: CD8^+^; **(I)**: CD4^+^/CD8^+^.

A total of 29 studies (Chen, 2008; Huang et al., 2010; Ning, 2011; Pan, 2011; Qiu et al., 2012; Ai et al., 2013; Ren et al., 2014; Shen et al., 2014; Zheng and Pan, 2014; Zhou, 2014; Chen et al., 2015; Zhang Z. Y. et al., 2015; Huang, 2015; Yao, 2015; Cui and Dai, 2016; Wang and Wu, 2016; Zhang, 2016; Jin et al., 2017; Lu et al., 2017; Zhang, 2017; Zhang et al., 2017; Zhu, 2017; Cheng et al., 2018; Ma et al., 2018; Feng, 2019; Li. X et al., 2019; Meiling Li et al., 2019; Zhang, 2019; Zhang and Zhang, 2019) that were compared to six treatments were included in this analysis. Shenfu injection combined with WM was used frequently to assess APACHE II score. There was significant heterogeneity among studies as shown in Table 2, so a random-effect model was employed to conduct network meta-analysis. As seen in Table 2, four CHIs investigated combined with WM were effective in improving APACHE II score except Shengmai injection when compared to WM alone: Huangqi vs. WM (MD = −5.80, 95% CI = −9.13 to −2.38); Shenqifuzheng vs. WM (MD = −4.72, 95% CI = −6.50 to −2.97); Shenmai vs. WM (MD = −3.10, 95% CI = −4.63 to −1.55); Shenfu vs. WM (MD = −2.49, 95% CI = −3.60 to −1.35). In addition, Shenqifuzheng combined with WM was more effective than Shenfu combined with WM (MD = −2.24, 95% CI = −4.35 to −0.16). Treatments ranking based on SUCRA values, which were shown in Figure 4 and Table 11, from largest to smallest, were as follows: Hangqi (91%), Shenqifuzheng (82.3%), Shenmai (52.8%), Shenfu (38.8%), Shengmai (32.8%) and WM (2.4%). Node splitting method results showed no inconsistency existing between direct and indirect evidence according to Supplementary Table S1. The funnel plot for APACHE II score was displayed in Figure 5 and showed significant asymmetry, which indicated possible publication bias.

**TABLE 2:** MDs with 95% CIs of APACHE II score. Significant effects are printed in bold.

HQ + WM					*p* = 0.007, *I* ^*2*^ = 86%
−1.08 (−4.83, 2.79)	**SQFZ + WM**				*p <* 0.00001, *I* ^*2*^ = 94%
−2.70 (−6.37, 1.05)	−1.62 (−3.98, 0.71)	**SM + WM**			*p <* 0.00001, *I* ^*2*^ = 87%
−3.31 (−6.83, 0.29)	−**2.24 (**−**4.35,** −**0.16)**	−0.61 (−2.52, 1.29)	**SF + WM**		*p <* 0.0001, *I* ^*2*^ = 71%
−3.84 (−8.54, 0.95)	−2.77 (−6.55, 1.01)	1.14 (−2.53, 4.83)	0.53 (−2.82, 3.87)	**SGM + WM**	
−**5.80 (**−**9.13,** −**2.38)**	−**4.72 (**−**6.50,** −**2.97)**	−**3.10 (**−**4.63,** −**1.55)**	−**2.49 (**−**3.60,** −**1.35)**	−1.95 (−5.28, 1.4)	**WM**

### Secondary Outcomes

#### 28-days Mortality

13 studies (Zhang et al., 2011; Ren et al., 2014; Zheng and Pan, 2014; Zhou, 2014; Huang, 2015; Zhou et al., 2015; Cui and Dai, 2016; Huang, 2016; Lei and Li, 2016; Zhang et al., 2017; Zhu, 2017; Meiling Li et al., 2019; Zhang, 2019) with four treatments including Shenfu, Shengmai, Huangqi and WM alone reported the 28-days mortality. There was no heterogeneity among studies as shown in Table 3. Shenfu combined WM was more effective than WM alone (OR = 0.55, 95% CI = 0.37 to 0.78), while the results showed no significant difference in the remaining cases according to Table 3. Treatments ranking based on SUCRA values, which were shown in Figure 4 and Table 11, from largest to smallest, were as follows: Shenfu (74.8%), Shengmai (63.3%), Huangqi (43.5%) and WM (18.4%). Node splitting method results and funnel plot were shown in Supplementary Table S2 and Figure 5.

**TABLE 3:** ORs with 95% CIs of 28-days mortality. Significant effects are printed in bold.

SF + WM			*p* = 0.79, *I* ^*2*^ = 0%
1.29 (0.34, 3.38)	**SGM + WM**		
1.99 (0.32, 6.70)	2.17 (0.21, 8.89)	**HQ + WM**	
**0.55 (0.37, 0.78)**	0.69 (0.18, 1.81)	1.05 (0.18, 3.45)	**WM**

### PCT

19 studies (Ai et al., 2013; Liu and Zhang, 2013; Ren et al., 2013; Qin, 2014; Chen et al., 2015; Huang, 2015; Xu et al., 2015; Wang and Wu, 2016; Zhou et al., 2016; Lu et al., 2017; Zhang and Wang, 2017; Zhu, 2017; Cheng et al., 2018; Liu et al., 2018; Liu and Yang, 2018; Yan, 2018; Zhang, 2019; Chen. T. F. et al., 2020; Pan and Chen, 2020) with six treatments reported the PCT. There was significant heterogeneity among studies as shown in Table 4. Three CHIs investigated combined with WM were effective in reducing the level of PCT when compared to WM alone: Shenmai vs. WM (SMD = −2.44, 95% CI = −3.23 to −1.65); Shengmai vs. WM (SMD = −1.80, 95% CI = −2.87 to −0.72); Shenfu vs. WM (SMD = −1.62, 95% CI = −2.27 to −0.98) according to Table 4. Treatments ranking based on SUCRA values, which were shown in Figure 4 and Table 11, from largest to smallest, were as follows: Shenmai (94.4%), Shengmai (71.9%), Shenfu (64.6%), Shenqifuzheng (38%), WM (17.4%) and Huangqi (13.8%). Node splitting method results and funnel plot were shown in Supplementary Table S3 and Figure 5.

**TABLE 4:** SMDs with 95% CIs of PCT. Significant effects are printed in bold.

SM + WM				*p* < 0.00001, *I* ^*2*^ = 95%	
0.65 (−0.69, 1.98)	**SGM + WM**				
−0.82 (−1.84, 0.20)	−0.17 (−1.34, 1.01)	**SF + WM**		*p <* 0.00001, *I* ^*2*^ = 93%	
1.77 (−0.32, 3.87)	1.13 (−1.09, 3.35)	0.95 (−1.09, 3.0)	**SQFZ + WM**		
−**2.44 (**−**3.23,** −**1.65)**	−**1.80 (**−**2.87,** −**0.72)**	−**1.62 (**−**2.27,** −**0.98)**	−0.67 (−2.61, 1.27)	**WM**	
2.75 (0.65, 4.85)	2.11 (−0.12, 4.32)	1.93 (−0.12, 3.98)	0.98 (−1.77, 3.72)	0.31 (−1.63, 2.25)	**HQ + WM**

### CRP

Seventeen studies (Qiu et al., 2012; Ai et al., 2013; Liu and Zhang, 2013; Ren et al., 2013; Qin, 2014; Chen et al., 2015; Xu et al., 2015; Wang and Wu, 2016; Lu et al., 2017; Zhu, 2017; Li, 2018; Liu and Yang, 2018; Feng, 2019; Zhang, 2019; Chen. T. F. et al., 2020; Pan and Chen, 2020; Li et al., 2021) with six treatments reported the CRP. There was significant heterogeneity among studies as shown in Table 5. Three CHIs investigated combined with WM were superior in reducing the level of CRP when compared to WM alone: Shenmai vs. WM (SMD = −3.22, 95% CI = −4.02 to −2.41); Shenqifuzheng vs. WM (SMD = −2.66, 95% CI = −4.06 to −1.26); Shengmai vs. WM (SMD = −1.15, 95% CI = −2.25 to −0.04) according to Table 5. What’s more, based on WM, Shenmai and Shenqifuzheng had more excellent performance in decreasing CRP than Shenfu. Treatments ranking based on SUCRA values, which were shown in Figure 4 and Table 11, from largest to smallest, were as follows: Shenmai (94.8%), Shenqifuzheng (83.1%), Shengmai (51.4%), Huangqi (31.2%), Shenfu (30.9%) and WM (8.5%). Node splitting method results and funnel plot were shown in Supplementary Table S4 and Figure 5.

**TABLE 5:** SMDs with 95% CIs of CRP. Significant effects are printed in bold.

SM + WM					*p <* 0.0001, *I* ^*2*^ = 98%
0.56 (−1.06, 2.17)	**SQFZ + WM**				*p* = 0.0006, *I* ^*2*^ = 94%
2.07 (0.70, 3.44)	−1.51 (−3.30, 0.27)	**SGM + WM**			*p <* 0.00001, *I* ^*2*^ = 96%
2.68 (0.54, 4.82)	2.13 (−0.30, 4.56)	0.61 (−1.66, 2.88)	**HQ + WM**		
−**2.71 (**−**3.85,** −**1.58)**	−**2.15 (**−**3.77,** −**0.54)**	−0.64 (−1.91, 0.62)	−0.03 (−2.17, 2.10)	**SF + WM**	*p <* 0.0001, *I* ^*2*^ = 91%
−**3.22 (**−**4.02,** −**2.41)**	−**2.66 (**−**4.06,** −**1.26)**	−**1.15 (**−**2.25,** −**0.04)**	−0.53 (−2.51, 1.45)	−0.50 (−1.30, 0.29)	**WM**

### IL-6

IL-6 was estimated in 14 studies (Chen, 2008; Huang et al., 2010; Qiu et al., 2012; Ren et al., 2013; Zhang Z. Y. et al., 2015; Xu et al., 2015; Zhou et al., 2015; Wang et al., 2016; Jin et al., 2017; Cheng et al., 2018; Yan, 2018; Feng, 2019; Meiling Li et al., 2019; Li et al., 2021) with six treatments. There was significant heterogeneity among studies as shown in Table 6. Four CHIs investigated combined with WM were outstanding in decreasing the level of PCT when compared to WM alone: Shenfu vs. WM (SMD = −4.41, 95% CI = −5.23 to −3.59); Shengmai vs. WM (SMD = −2.26, 95% CI = −4.27 to −0.24); Shenmai vs. WM (SMD = −2.05, 95% CI = −3.21 to −0.89); Shenqifuzheng vs. WM (SMD = −1.46, 95% CI = −2.89 to −0.02) according to Table 6. Treatments ranking based on SUCRA values, which were shown in Figure 4 and Table 11, from largest to smallest, were as follows: Shenfu (99.5%), Shengmai (64.2%), Shenmai (61.8%), Shenqifuzheng (45.5%), Huangqi (24.8%) and WM (4.1%). Funnel plot were shown in Figure 5.

**TABLE 6:** SMDs with 95% CIs of IL-6. Significant effects are printed in bold.

SF + WM					*p <* 0.00001, *I* ^*2*^ = 95%
2.15 (−0.03, 4.33)	**SGM + WM**				
2.36 (0.94, 3.78)	−0.21 (−2.53, 2.12)	**SM + WM**			*p* = 0.004, *I* ^*2*^ = 82%
2.95 (1.30, 4.61)	0.80 (−1.67, 3.28)	0.59 (−1.25, 2.44)	**SQFZ + WM**		*p* = 0.0003, *I* ^*2*^ = 92%
3.71 (2.07, 5.35)	1.56 (−0.90, 4.03)	1.36 −0.48, 3.19)	0.76 (−1.26, 2.79)	**HQ + WM**	*p* = 0.94, *I* ^*2*^ = 0%
−**4.41 (**−**5.23,** −**3.59)**	−**2.26 (**−**4.27,** −**0.24)**	−**2.05 (**−**3.21,** −**0.89)**	−**1.46 (**−**2.89,** −**0.02)**	−0.69 (−2.12, 0.73)	**WM**

### TNF-α

Twenty studies (Chen, 2008; Huang et al., 2010; Liu and Zhang, 2013; Qin, 2014; Chen et al., 2015; Zhang Z. Y. et al., 2015; Xu et al., 2015; Zhou et al., 2015; Hu et al., 2016; Wang et al., 2016; Wang and Wu, 2016; Zhao et al., 2016; Jin et al., 2017; Cheng et al., 2018; Liu and Yang, 2018; Yan, 2018; Feng, 2019; Li et al., 2019; Li et al., 2021; Ning et al., 2012) with six treatments reported the TNF-α. There was significant heterogeneity among studies as shown in Table 7. Four CHIs investigated combined with WM were excellent in reducing the level of TNF-α when compared to WM alone: Shenfu vs. WM (SMD = −4.02, 95% CI = −4.85 to −3.20); Shengmai vs WM (SMD = −2.65, 95% CI = −3.66 to −1.65); Shenmai vs WM (SMD = −2.45, 95% CI = −3.26 to −1.63); Shenqifuzheng vs WM (SMD = −1.93, 95% CI = −3.36 to −0.50) according to Table 7. Treatments ranking based on SUCRA values, which were shown in Figure 4 and Table 11, from largest to smallest, were as follows: Shenfu (99.1%), Shengmai (68.7%), Shenmai (62.2%), Shenqifuzheng (49.5%), WM (16.8%) and Huangqi (3.4%). Funnel plot were shown in Figure 5.

**TABLE 7:** SMDs with 95% CIs of TNF-α. Significant effects are printed in bold.

SF + WM				*p* < 0.00001, *I* ^*2*^ = 95%	
1.37 (0.07, 2.67)	**SGM + WM**			*p* = 0.04, *I* ^*2*^ = 70%	
1.58 (0.42, 2.74)	−0.21 (−1.50, 1.09)	**SM + WM**		*p* < 0.00001, *I* ^*2*^ = 97%	
2.10 (0.44, 3.74)	0.72 (−1.03, 2.47)	0.52 (−1.13, 2.17)	**SQFZ + WM**	*p* < 0.00001, *I* ^*2*^ = 96%	
**−4.02 (−4.85, −3.20)**	**−2.65 (−3.66, −1.65)**	**−2.45 (−3.26, −1.63)**	**−1.93 (−3.36, −0.50)**	**WM**	*P* = 0.0002, *I* ^*2*^ = 93%
4.74 (3.09, 6.38)	3.37 (1.63, 5.11)	3.16 (1.51, 4.80)	2.64 (0.62, 4.66)	0.71 (−0.71, 2.14)	**HQ + WM**

### CD4^+^

Eleveen studies (Chen, 2008; Qiu et al., 2012; Ai et al., 2013; Shen et al., 2014; Chen et al., 2015; Zhang Z. Y. et al., 2015; Zhang and Wang, 2017; Zhu, 2017; Li, 2018; Liu and Yang, 2018; Ma et al., 2018) with five treatments reported the CD4^+^. There was significant heterogeneity among studies as shown in Table 8. Three CHIs investigated combined with WM were effective in increasing the level of CD4^+^ when compared to WM alone: Huangqi vs. WM (SMD = 1.92, 95% CI = 0.21 to 3.63); Shenqifuzheng vs. WM (SMD = 1.28, 95% CI = 0.42 to 2.14); Shenmai vs. WM (SMD = 1.26, 95% CI = 0.27 to 2.25) according to Table 8. Treatments ranking based on SUCRA values, which were shown in Figure 4 and Table 11, from largest to smallest, were as follows: Huangqi (83.3%), Shenqifuzheng (62.1%), Shenmai (60.6%), Shenfu (42.5%), and WM (1.5%). Funnel plot were shown in Figure 5.

**TABLE 8:** SMDs with 95% CIs of CD4^+^. Significant effects are printed in bold.

HQ + WM				
0.63 (−1.28, 2.55)	**SQFZ + WM**			*p* = 0.56, *I* ^*2*^ = 0%
0.66 (−1.32, 2.64)	0.02 (−1.29, 1.34)	**SM + WM**		*p <* 0.0001, *I* ^*2*^ = 91%
1.01 (−0.97, 2.99)	0.38 (−0.94, 1.70)	0.36 (−1.06, 1.77)	**SF + WM**	*p* = 0.0002, *I* ^*2*^ = 88%
**1.92 (0.21, 3.63)**	**1.28 (0.42, 2.14)**	**1.26 (0.27, 2.25)**	0.91 (−0.10, 1.91)	**WM**

### CD8^+^

Ten studies (Chen, 2008; Qiu et al., 2012; Ai et al., 2013; Shen et al., 2014; Chen et al., 2015; Zhang Z. Y. et al., 2015; Zhu, 2017; Li, 2018; Liu and Yang, 2018; Ma et al., 2018) with five treatments reported the CD8^+^. There was significant heterogeneity among studies as shown in Table 9. Two CHIs investigated combined with WM were effective in improving the level of CD8^+^ when compared to WM alone: Huangqi vs. WM (SMD = −2.77, 95% CI = −5.01 to −0.53); Shenqifuzheng vs. WM (SMD = −2.02, 95% CI = −3.60 to −0.43) according to Table 9. In addition, based on WM, Huangqi and Shenqifuzheng had more excellent performance in decreasing CD8^+^ than Shenfu. Treatments ranking based on SUCRA values, which were shown in four and Table 11, from largest to smallest, were as follows: Huangqi (92.2%), Shenqifuzheng (78.7%), WM (49.3%), Shenfu (23%), and Shenmai (6.9%). Funnel plot were shown in Figure 5.

**TABLE 9:** SMDs with 95% CIs of CD8^+^. Significant effects are printed in bold.

HQ + WM				
−0.76 (−2.80, 1.29)	**SQFZ + WM**	*p <* 0.00001, *I* ^*2*^ = 91%		
−1.50 (−3.34, 0.33)	−0.74 (−1.65, 0.16)	**WM**	*p <* 0.00001, *I* ^*2*^ = 92%	*p <* 0.00001, *I* ^*2*^ = 97%
−**2.17 (**−**4.28,** −**0.05)**	−**1.41 (**−**2.81,** −**0.01)**	0.66 (−0.40, 1.73)	**SF + WM**	
−**2.77 (**−**5.01,** −**0.53)**	−**2.02 (**−**3.60,** −**0.43)**	1.27 (−0.02, 2.56)	0.61 (−1.07, 2.28)	**SM + WM**

### CD4^+^/CD8^+^

Ten studies (Chen, 2008; Qiu et al., 2012; Ai et al., 2013; Shen et al., 2014; Chen et al., 2015; Zhang Z. Y. et al., 2015; Zhang and Wang, 2017; Zhu, 2017; Li, 2018; Ma et al., 2018) with five treatments reported the CD4^+^/CD8^+^. There was significant heterogeneity among studies as shown in Table 10. Two CHIs investigated combined with WM were effective in improving the CD4^+^/CD8^+^ when compared to WM alone: Huangqi vs. WM (MD = 0.86, 95% CI = 0.17 to 1.55); Shenqifuzheng vs. WM (MD = 0.38, 95% CI = 0.04 to 0.71) according to Table 10. Treatments ranking based on SUCRA values, which were shown in Figure 4 and Table 11, from largest to smallest, were as follows: Huangqi (93.6%), Shenmai (59.1%), Shenqifuzheng (55.9%), Shenfu (35.9%), and WM (5.4%). Funnel plot were shown in Figure 5.

**TABLE 10:** SMDs with 95% CIs of CD4^+^/CD8^+^. Significant effects are printed in bold.

HQ + WM				
0.45 (−0.39, 1.32)	**SM + WM**			*p* = 0.27, *I* ^*2*^ = 17%
0.48 (−0.28, 1.25)	−0.03 (−0.63, 0.59)	**SQFZ + WM**		*p <* 0.00001, *I* ^*2*^ = 93%
0.64 (−0.19, 1.45)	0.18 (−0.51, 0.84)	0.15 (−0.42, 0.71)	**SF + WM**	*p* = 0.12, *I* ^*2*^ = 53%
**0.86 (0.17, 1.55)**	0.41 (−0.11, 0.90)	**0.38 (0.04, 0.71)**	0.23 (−0.22, 0.68)	**WM**

**TABLE 11:** SUCRA results of the outcomes.

	APACHE II score (%)	28-days mortality (%)	PCT (%)	CRP (%)	IL-6 (%)	TNF-α (%)	CD4^+^ (%)	CD8^+^ (%)	CD4^+^/CD8^+^ (%)
WM	2.4	18.4	17.4	8.5	4.1	16.8	1.5	49.3	5.4
SF + WM	38.8	74.8	64.6	30.9	99.5	99.4	42.5	23	35.9
SM + WM	52.8	—	94.4	94.8	61.8	62.2	60.6	6.9	59.1
SGM + WM	32.8	63.3	71.9	51.4	64.2	68.7	—	—	—
SQFZ + WM	82.3	—	38	83.1	45.5	49.5	62.1	78.7	55.9
HQ + WM	91	43.5	13.8	31.2	24.8	3.4	83.3	92.2	93.6

### ADRs/ADEs

Among the included 50 RCTs, a total of six RCTs reported the ADRs/ADEs of CHIs. There were two studies (Zhang, 2019; Li et al., 2021) involved six participants in Shenfu group associated with ADRs/ADEs, including headache and dizziness (three cases in two studies), nausea and vomiting (two cases in one study), diarrhea (one case in one study). Two studies (Ren et al., 2013; Ren et al., 2014) reported ADRs/ADEs of Huangqi injection, both of them occurred one case of rash and one case of diarrhea. Another two studies (Qin, 2014; Zhang, 2019) with Shengmai injection reported two cases of ADRs/ADEs, one case of flatulence and one case of diarrhea. The rest of included studies did not provide information on any ADRs/ADEs. All of the symptoms were alleviated by rest or symptomatic treatment without affecting the RCTs’ results.

### Sensitivity Analysis

There was significant heterogeneity between studies for the primary outcome. Hence, a sensitivity analysis was conducted for the outcome of APACHE II score. After omitting six studies (Chen, 2008; Pan, 2011; Ai et al., 2013; Shen et al., 2014; Wang and Wu, 2016; Zhang and Zhang, 2019), the *I* ^*2*^ values for standard pairwise meta-analysis were reduced obviously and all less than 50% according to Table 12. The remaining 23 studies were conducted a network meta-analysis again. The pooled MD and SUCRA value of Huangqi injection were changed significantly, while the rest CHIs were slightly modified when the individual study data were removed, one at a time, from any pairwise comparison analysis. The Bayesian ranking results of sensitivity analysis from largest to smallest were Shenqifuzheng (95.65%), Shenmai (74%), Shenfu (47.1%), Shengmai (35.3%), Huangqi (33.2%) and WM (3.4%), respectively.

**TABLE 12:** MDs with 95% CIs of APACHE II score. Significant effects are printed in bold.

SQFZ + WM					*p* = 0.29, *I* ^*2*^ = 20%
−1.10 (−2.58, 0.51)	**SM + WM**				*p* = 0.16, *I* ^*2*^ = 39%
−**2.10 (**−**3.41,** −**0.71)**	−1.00 (−2.22, 0.18)	**SF + WM**			*p* = 0.10, *I* ^*2*^ = 34%
−**2.58 (**−**4.48,** −**0.55)**	1.48 (−0.38, 3.37)	0.48 (−1.13, 2.06)	**SGM + WM**		
2.39 (−1.9, 6.64)	1.28 (−2.95, 5.53)	0.28 (−3.88, 4.45)	−0.19 (−4.59, 4.21)	**HQ + WM**	
−**4.48 (**−**5.59,** −**3.24)**	−**3.38 (**−**4.38,** −**2.39)**	−**2.38 (**−**3.03,** −**1.70)**	−**1.90 (**−**3.47,** −**0.31)**	−2.10 (−6.21, 2.02)	**WM**

## Discussion

A total of 50 studies involving 3,394 participants were included. Five tonic CHIs were identified in the treatment of sepsis or septic shock, including Shenfu injection, Shenmai injection, Shengmai injection, Shenqifuzheng injection, and Huangqi injection. According to the results of this NMA and sensitivity analysis, four CHIs including Shenqifuzheng injection, Shenmai injection, Shenfu injectuion and Shengmai injection combined with WM had a superior effect in improving the APACHE II score than WM alone and the differences were statistically significant. Based on sensitivity analysis and SUCRA values, Shenqifuzheng injection (95.65%) combined with WM ranked highest, followed by Shenmai injection (74%), Shenfu injection (47.1%), Shengmai injection (35.3%) and Huangqi injection (33.2%). Among the secondary outcomes, Shenmai injection was the most favorable intervention in reducing PCT and CRP levels, and Shenqifuzheng injection was the second favorable intervention in reducing CRP level. Shenfu injection combined with WM was more effective than the other treatments in decreasing the serum IL-6 and TNF-α levels and lowering the 28-days mortality. Regarding the improvement of immune function, Shenqifuzheng injections had obvious advantages.

As for safety, a total of six RCTs reported the ADRs/ADEs of CHIs, including two studies of Shenfu injection, two studies of Huangqi injection, and two studies of Shengmai injection. The ADRs/ADEs mainly involved headache, dizziness, nausea, vomiting, diarrhea, rash, and flatulence. Though all the ADRs/ADEs were mild and can be relieved by themselves, no studies reported the rate of ADRs/ADEs comparing CHIs combined with WM and WM alone. Hence, we could not draw a certain conclusion that combing CHIs with WM will not increase the ADRs/ADEs of the patients. Hopefully, further studies especially clinical trials should pay more attention to these ADRs/ADEs of CHIs and more studies are needed to determine the safety of CHIs combined with WM for sepsis.

The pathophysiology of sepsis is extremely complex. The causative pathogen produces an excessive inflammatory response with high levels of anti-inflammatory cytokines. These high levels of anti-inflammatory cytokines are associated with ICU admission and mortality. Finally, the early proinflammatory state in sepsis often develops into a later and prolonged state of immune system dysfunction over time (Gotts and Matthay, 2016).

Our study has found that Shenqifuzheng injection combined with WM had obvious advantages in improving APACHE II score, reducing CRP level, and especially enhancing immune function. Shenqifuzheng injection is a well-known Chinese traditional medicine to invigorate “Qi” and strengthen health, which is made of Codonopsis pilosula and Astragali Radix. The main active component of Codonopsis pilosula is Codonopsis pilosula polysaccharide. The related studies have demonstrated that polysaccharide isolated from Codonopsis pilosula have obvious immune-modulation effects (Zheng et al., 2014; Fu et al., 2018; Zou et al., 2019). Moreover, the polysaccharide could exhibit anti-inflammatory effect against lipopolysaccharide (LPS) induced RAW264.7 cells *in vitro* and *in vivo* and reduce the expression of inflammatory factors (Meng et al., 2020). Astragali Radix contains numerous natural products with different structural patterns and the main active constituents are astragalus polysaccharides, astragalus saponins and astragalus flavonoids. These main active constituents have shown considerable immunomodulatory properties both *in vitro* and *in vivo* (Gong et al., 2018; Chen Z. et al., 2020).

Another result of this study suggested that Shenfu injection combined with WM exhibited a better performance in reducing 28-days mortality and inhibiting inflammatory indicators, which were consistent with previous meta-analysis (Huang et al., 2019; Yu et al., 2019; Zhao et al., 2020). Shenfu injection is composed with Radix Ginseng Rubra and Radix Aconiti Lateralis Praeparata, which has great effect of restoring “Yang” from collapse and tonifying “Qi” for relieving desertion. Ginsenoside and aconitine are the main active ingredients in Shenfu injection. Modern pharmacological research shows that ginsenoside can suppress production of multiple inflammatory mediators such as TNF-α, interleukin (IL)-1β, IL-6, cyclooxygenase-2 (COX-2) and inducible nitric oxide synthase (iNOS) in Lipopolysaccharide (LPS)-stimulated cells, inhibit LPS-induced in body temperature, serum TNF-α, IL-1β, IL-6, COX-2, iNOS in rats, attenuate lethal sepsis, and protect mice from death in a mouse model of endotoxin shock (Su et al., 2012; Su et al., 2015). In addition, ginsenoside has dual roles in regulation of the immune responses: up-regulation of the immune responses and down-regulation of the proinflammatory response (Sun et al., 2008). Evidence has also revealed that aconitine has the effects of anti-inflammation and regulating the immune function. Researches have found that aconitine could partly inhibit the proliferation and NO production in LPS-induced RAW264.7 cells and showed anti-inflammatory effect by inhibiting macroscopic pathology and histological inflammation (Mi et al., 2021).

There are three advantages that could enhance the prestige of this study. First, to the best of our knowledge, this is the first NMA to compare the effects of different CHIs and rank them for the treatment of sepsis or septic shock. Secondly, these results may be helpful to clinicians to make a better choice for the treatment of sepsis or septic shock. Additionally, the inclusion and exclusion criteria were strictly established.

### Limitations

This study also has some limitations. First, all studies except one were published in China, and the data of clinical studies in other languages was lacking. Second, the qualities of included studies in this study were not high. Only three studies mentioned the method of allocation concealment. Third, there was a lack of large-sample direct comparisons between the two injections. The difference among the sample sizes of different injections would also reduce the strength of evidence for the results.

## Conclusion

In conclusion, Shenqifuzheng injection was the optimum treatment regimen to improve APACHE II score, reduce CRP level, and regulate immune function. Shenfu injection was superior in reducing the expression of inflammatory factors and decreasing 28-days mortality. Nevertheless, more multicenter, diverse, and direct comparisons RCTs are needed to further confirm the results.

## Data Availability

The original contributions presented in the study are included in the article/Supplementary Material, further inquiries can be directed to the corresponding authors.

## Abbreviations

CHI, Chinese herbal injection; NMA, network meta-analysis; SUCRA: surface under the cumulative ranking curve; RCT: randomized controlled trial; AMR: antimicrobial resistance; AKI: acute kidney injury; ICU: intensive care unit; WM: Western medicine; OR: odds ratio; MD: mean difference; SMD: standardized mean difference; ADRs/ADEs: adverse drug reactions or adverse drug events; CNKI: China National Knowledge Infrastructure; VIP: Chinese Scientific Journal Database; SF: Shenfu injection; SM: Shenmai injection; SGM: Shengmai injection; SQFZ: Shenqifuzheng injection; HQ: Huangqi injection.
