# Supplementary material for: Comparative Efficacy of Tonic Chinese Herbal Injections for Treating Sepsis or Septic Shock: A Systematic Review and Bayesian Network Meta-Analysis of Randomized Controlled Trials
Source: Front Pharmacol. 2022 Mar 15;13:830030. doi: 10.3389/fphar.2022.830030 (PMC8972587; doi:10.3389/fphar.2022.830030)
Supplement: Supplementary file 2 [file DataSheet3.docx]

More details about the product information of 5 CHIs

| Injection name | Source | Species/Raw materials | Component ingredients to be measured | Botanical plant names | Indications | Adverse drug reactions | Quality control reported? (Y/N) | Chemical analysis reporter? (Y/N) |
| --- | --- | --- | --- | --- | --- | --- | --- | --- |
| Shenfu injection | Ya'an Sanjiu Pharmaceutical Co., Ltd. | Red Ginseng, *Aconitum carmichaeli Debeaux* | Ginsenoside, >0.5mg/mL;  aconitine, <0.1mg/mL | *Panax ginseng* C.A.Mey.; *Aconitum carmichaeli Debeaux* | Desertion syndrome caused by excessive yang-qi insufficiency (infectious, haemorrhagic and fluid loss shock); yang or qi deficiencies with palpitations, cough, stomach ache, diarrhoea, rheumatism, etc. | Pruritus, skin rash, diarrhea, nausea, vomit, anaphylactic shock | Y - WB_3_-B-3427-98-2013 issued by China Food and Drug Administration | N |
| Shenmai injection | Ya'an Sanjiu Pharmaceutical Co., Ltd.; Zhengda Qingchunbao Pharmaceutical Co., Ltd.; Hebei Shenwei Pharmaceutical Co., Ltd.; Sichuan Chuanda West China Pharmaceutical Co., Ltd.; Yunnan Gejiu Biopharmaceutical Co., Ltd.; Sichuan Shenghe Pharmaceutical Co., Ltd.; Dali Pharmaceutical Co., Ltd. | Red Ginseng, Radix Ophiopogonis | Ginsenoside, Panaxatriol, Ophiopogonone | *Panax ginseng* C.A.Mey.; *Ophiopogon japonicus* (Thunb.) Ker Gawl | Shock, coronary heart disease, viral myocarditis, chronic pulmonary heart disease and neutropenia with qi and yin deficiencies; improves immune function of patients with tumours, combined with chemotherapy to enhance curative effects and to reduce toxic and side effects, etc. | Dizziness, diarrhea, nausea, vomit, pruritus, chest congestion, skin rash, fever, edema, phlebitis, anaphylactic shock, dyspnea, palpitation, xerostomia | Y - WS_3_-B-3428-98-2010 issued by China Food and Drug Administration | N |
| Shengmai injection | Jiangsu Suzhong Construction Group Co., Ltd.; Changshu LEI YUN SHANG Pharmaceutical Co., Ltd.; Shanxi TAIHANG Pharmaceutical Co., Ltd. | Red Ginseng, Ophiopogonis, *Schisandrae Chinensis Fructus* | Ginsenoside, Panaxatriol, Ophiopogonone, Methylophiopogonanone, Shikimic Acid and et al. | *Panax ginseng* C.A.Mey.; *Ophiopogon japonicus* (Thunb.) Ker Gawl; *Schisandra chinensis* (Turcz.) Baill. | Palpitations, hard breathing, cold limbs and other symptoms caused by qi and yin insufficiencies, myocardial infarction, cardiogenic shock, cardiogenic shock and others with abovementioned symptoms, etc. | skin rash, dyspnea, Pruritus, anaphylactic shock, palpitation; fever, chilliness; diarrhea, nausea, vomit, xerostomia; chest congestion, arrhythmia; phlebitis | Y - WS_3_-B-2865-98-2011 issued by China Food and Drug Administration | N |
| Shenqifuzheng injection | LIVZON Pharmaceutical Group Co., Ltd. | *Astragali Radix*, *Codonopsis Radix* | Codonopsis pilosula polysaccharide, astragalus polysaccharides, astragalus saponins and astragalus flavonoids | *Astragalus mongholicus* Bunge; *Codonopsis pilosula* (Franch.) Nannf. | Fatigue, lack of strength, vertigo caused by asthenia of pulmonosplenic qi; auxiliary treatment of the above symptoms for lung cancer and gastric cancer, etc. | mild bleeding, fever, xerostomia, hypersomnia, skin rash, chilliness, chest congestion, vomit, palpitation | Y - National Drug Standard WS3-387（Z-50）- 2003（Z）-2001 and National Pharmaceutical Standard Z19990065 issued by China Food and Drug Administration | N |
| Huangqi injection | Chengdu Diao Pharmaceutical group Co., Ltd.; Zhengda Qingchunbao Pharmaceutical Co., Ltd.; Shanghai Fuda Pharmaceutical Drug manufacturing Co., Ltd. | *Astragali Radix* | Astragaloside, Isoastragaloside, Acetylastragaloside, Astragaline, Benzofiiranoidlignan and et al. | *Astragalus mongholicus* Bunge | Insufficiency of the heart-qi and blood stasis syndrome of viral myocarditis, cardiac insufficiency and et al.; hepatitis with spleen deficiency and dampness syndrome, etc. | skin rash, fever, anaphylactic shock; dyspnea, larynx edema, chest congestion; phlebitis, rapid atrial fibrillation; diarrhea, vomit, headache | Y - WS_3_-B-3335-98 issued by China Food and Drug Administration | N |

Detailed information about the CHIs used in the included studies

| Study | Chinese herbal injection | Source | Species | Quality control reported? (Y/N) | Chemical analysis reported? (Y/N) |
| --- | --- | --- | --- | --- | --- |
| Zhang N 2011 | Shenfu injection | Not mentioned | *Panax ginseng* C.A.Mey.[Araliaceae]; *Aconitum carmichaeli Debeaux* [Ranunculaceae] | N | N |
| Pan XN 2011 | Shenqifuzheng injection | LIVZON Pharmaceutical Group Co., Ltd. | *Astragalus mongholicus* Bunge [Fabaceae]; *Codonopsis pilosula* (Franch.) Nannf [Campanulaceae]. | N | N |
| Qiu ZL 2012 | Shenfu injection | Not mentioned | *Panax ginseng* C.A.Mey.[Araliaceae]; *Aconitum carmichaeli Debeaux* [Ranunculaceae] | N | N |
| Zheng Y 2014 | Shenfu injection | Ya'an Sanjiu Pharmaceutical Co., Ltd. | *Panax ginseng* C.A.Mey.[Araliaceae]; *Aconitum carmichaeli Debeaux* [Ranunculaceae] | Y - Z20043116 issued by China Food and Drug Administration | N |
| Ren Y 2014 | Huangqi injection | Not mentioned | *Astragalus mongholicus* Bunge [Fabaceae] | N | N |
| Qin HF 2014 | Shengmai injection | Jiangsu Suzhong Construction Group Co., Ltd. | *Panax ginseng* C.A.Mey.[Araliaceae]; *Ophiopogon japonicus* (Thunb.) Ker Gawl [Asparagaceae]; *Schisandra chinensis* (Turcz.) Baill. [Schisandra chinensis] | Y - Z32021056 issued by China Food and Drug Administration | N |
| Yao S 2015 | Shenfu injection | Not mentioned | *Panax ginseng* C.A.Mey.[Araliaceae]; *Aconitum carmichaeli Debeaux* [Ranunculaceae] | N | N |
| Zhang ZY 2015 | Shenqifuzheng injection | LIVZON Pharmaceutical Group Co., Ltd. | *Astragalus mongholicus* Bunge [Fabaceae]; *Codonopsis pilosula* (Franch.) Nannf [Campanulaceae]. | N | N |
| Huang MH 2015 | Shenfu injection | Not mentioned | *Panax ginseng* C.A.Mey.[Araliaceae]; *Aconitum carmichaeli Debeaux* [Ranunculaceae] | N | N |
| Liu PF 2018 | Shenmai injection | Ya'an Sanjiu Pharmaceutical Co., Ltd. | *Panax ginseng* C.A.Mey.[Araliaceae]; *Ophiopogon japonicus* (Thunb.) Ker Gawl. [Asparagaceae] | Y - Z51020552 issued by China Food and Drug Administration | N |
| Li XX 2021 | Shenfu injection | Ya'an Sanjiu Pharmaceutical Co., Ltd. | *Panax ginseng* C.A.Mey.[Araliaceae]; *Aconitum carmichaeli Debeaux* [Ranunculaceae] | Y - China lot number: 20181225 | N |
| Zhou HF 2016 | Shenfu injection | Ya'an Sanjiu Pharmaceutical Co., Ltd. | *Panax ginseng* C.A.Mey.[Araliaceae]; *Aconitum carmichaeli Debeaux* [Ranunculaceae] | Y - China lot number: 141004050 | N |
| Wang S 2016 | Shenmai injection | Hebei Shenwei Pharmaceutical Co., Ltd. | *Panax ginseng* C.A.Mey.[Araliaceae]; *Ophiopogon japonicus* (Thunb.) Ker Gawl. [Asparagaceae] | Y - Z13020887 issued by China Food and Drug Administration | N |
| Zhao XF 2016 | Huangqi injection | Not mentioned | *Astragalus mongholicus* Bunge [Fabaceae] | N | N |
| Wang YF 2016 | Shengmai injection | Not mentioned | *Panax ginseng* C.A.Mey. [Araliaceae]; *Ophiopogon japonicus* (Thunb.) Ker Gawl [Asparagaceae]; *Schisandra chinensis* (Turcz.) Baill. [Schisandraceae] | N | N |
| Hu DC 2016 | Shengmai injection | Not mentioned | *Panax ginseng* C.A.Mey. [Araliaceae]; *Ophiopogon japonicus* (Thunb.) Ker Gawl [Asparagaceae]; *Schisandra chinensis* (Turcz.) Baill. [Schisandraceae] | N | N |
| Zhang SY 2017 | Shenfu injection | Ya'an Sanjiu Pharmaceutical Co., Ltd. | *Panax ginseng* C.A.Mey.[Araliaceae]; *Aconitum carmichaeli Debeaux* [Ranunculaceae] | Y - China lot number: 140807010 and 150705010 | N |
| Lu D 2017 | Shenmai injection | Zhengda Qingchunbao Pharmaceutical Co., Ltd. | *Panax ginseng* C.A.Mey.[Araliaceae]; *Ophiopogon japonicus* (Thunb.) Ker Gawl. [Asparagaceae] | Y - Z33020018 issued by China Food and Drug Administration | N |
| Jin LN 2017 | Shenqifuzheng injection | LIVZON Pharmaceutical Group Co., Ltd. | *Astragalus mongholicus* Bunge [Fabaceae]; *Codonopsis pilosula* (Franch.) Nannf [Campanulaceae]. | N | N |
| Zhu MG 2017 | Shenfu injection | Not mentioned | *Panax ginseng* C.A.Mey.[Araliaceae]; *Aconitum carmichaeli Debeaux* [Ranunculaceae] | N | N |
| Cheng TC 2018 | Shenfu injection | Ya'an Sanjiu Pharmaceutical Co., Ltd. | *Panax ginseng* C.A.Mey.[Araliaceae]; *Aconitum carmichaeli Debeaux* [Ranunculaceae] | Y - Z51020664 issued by China Food and Drug Administration | N |
| Liu Y 2018 | Shenfu injection | Ya'an Sanjiu Pharmaceutical Co., Ltd. | *Panax ginseng* C.A.Mey.[Araliaceae]; *Aconitum carmichaeli Debeaux* [Ranunculaceae] | Y - China lot number: 991201 | N |
| Yan ZJ 2018 | Shenfu injection | Ya'an Sanjiu Pharmaceutical Co., Ltd. | *Panax ginseng* C.A.Mey.[Araliaceae]; *Aconitum carmichaeli Debeaux* [Ranunculaceae] | N | N |
| Li P 2018 | Shenqifuzheng injection | LIVZON Pharmaceutical Group Co., Ltd. | *Astragalus mongholicus* Bunge [Fabaceae]; *Codonopsis pilosula* (Franch.) Nannf [Campanulaceae]. | Y - Z19990065 issued by China Food and Drug Administration | N |
| Li X 2019 | Shenfu injection | Ya'an Sanjiu Pharmaceutical Co., Ltd. | *Panax ginseng* C.A.Mey.[Araliaceae]; *Aconitum carmichaeli Debeaux* [Ranunculaceae] | Y - Z51020664 issued by China Food and Drug Administration | N |
| Li ML 2019 | Shenfu injection | Ya'an Sanjiu Pharmaceutical Co., Ltd. | *Panax ginseng* C.A.Mey.[Araliaceae]; *Aconitum carmichaeli Debeaux* [Ranunculaceae] | N | N |
| Pan Y 2020 | Shenfu injection | Ya'an Sanjiu Pharmaceutical Co., Ltd. | *Panax ginseng* C.A.Mey.[Araliaceae]; *Aconitum carmichaeli Debeaux* [Ranunculaceae] | Y - Z51020664 issued by China Food and Drug Administration | N |
| Lei XY 2016 | Shenfu injection | Ya'an Sanjiu Pharmaceutical Co., Ltd. | *Panax ginseng* C.A.Mey.[Araliaceae]; *Aconitum carmichaeli Debeaux* [Ranunculaceae] | Y - Z51020664 issued by China Food and Drug Administration | N |
| Zhou CL 2014 | Shenfu injection | Ya'an Sanjiu Pharmaceutical Co., Ltd. | *Panax ginseng* C.A.Mey.[Araliaceae]; *Aconitum carmichaeli Debeaux* [Ranunculaceae] | N | N |
| Chen RJ 2015 | Shenfu injection | Ya'an Sanjiu Pharmaceutical Co., Ltd. | *Panax ginseng* C.A.Mey.[Araliaceae]; *Aconitum carmichaeli Debeaux* [Ranunculaceae] | N | N |
| Zhou XL 2015 | Shenfu injection | Not mentioned | *Panax ginseng* C.A.Mey.[Araliaceae]; *Aconitum carmichaeli Debeaux* [Ranunculaceae] | N | N |
| Cui Y 2016 | Shenfu injection injection | Ya'an Sanjiu Pharmaceutical Co., Ltd. | *Panax ginseng* C.A.Mey.[Araliaceae]; *Aconitum carmichaeli Debeaux* [Ranunculaceae] | Y - Z20043117 issued by China Food and Drug Administration | N |
| Huang XX 2016 | Shenfu injection | Ya'an Sanjiu Pharmaceutical Co., Ltd. | *Panax ginseng* C.A.Mey.[Araliaceae]; *Aconitum carmichaeli Debeaux* [Ranunculaceae] | N | N |
| Zhang JL 2019 | Shenfu injection | Guangdong Xinfeng Pharmaceutical Co., Ltd. | *Panax ginseng* C.A.Mey.[Araliaceae]; *Aconitum carmichaeli Debeaux* [Ranunculaceae] | Y - Z51020664 issued by China Food and Drug Administration | N |
| Zhang XS 2019(a) | Shenfu injection | Not mentioned | *Panax ginseng* C.A.Mey.[Araliaceae]; *Aconitum carmichaeli Debeaux* [Ranunculaceae] | N | N |
| Zhang XS 2019(b) | Shengmai injection | Not mentioned | *Panax ginseng* C.A.Mey. [Araliaceae]; *Ophiopogon japonicus* (Thunb.) Ker Gawl [Asparagaceae]; *Schisandra chinensis* (Turcz.) Baill. [Schisandraceae] | N | N |
| Huang ZF 2010 | Shenmai injection | Hebei Shenwei Pharmaceutical Co., Ltd. | *Scutellaria baicalensis* Georgi [Lamiaceae], Selenarctos thibetanus Cuvier, Capra hircus Linnaeus, *Lonicera japonica* Thunb [Caprifoliaceae], *Forsythia suspensa* (Thunb.) Vahl [Oleaceae] | Y - Z13020887 issued by China Food and Drug Administration | N |
| Ning XP 2011 | Shenmai injection | Not mentioned | *Panax ginseng* C.A.Mey.[Araliaceae]; *Ophiopogon japonicus* (Thunb.) Ker Gawl. [Asparagaceae] | N | N |
| Ning XP 2012 | Shenmai injection | Not mentioned | *Panax ginseng* C.A.Mey.[Araliaceae]; *Ophiopogon japonicus* (Thunb.) Ker Gawl. [Asparagaceae] | N | N |
| Shen LM 2014 | Shenmai injection | Zhengda Qingchunbao Pharmaceutical Co., Ltd. | *Panax ginseng* C.A.Mey.[Araliaceae]; *Ophiopogon japonicus* (Thunb.) Ker Gawl. [Asparagaceae] | N | N |
| Xu XY 2015 | Shenmai injection | Zhengda Qingchunbao Pharmaceutical Co., Ltd. | *Panax ginseng* C.A.Mey.[Araliaceae]; *Ophiopogon japonicus* (Thunb.) Ker Gawl. [Asparagaceae] | N | N |
| Zhang L 2016 | Shenmai injection | Not mentioned | *Panax ginseng* C.A.Mey.[Araliaceae]; *Ophiopogon japonicus* (Thunb.) Ker Gawl. [Asparagaceae] | N | N |
| Zhang WM 2017 | Shenmai injection | Ya'an Sanjiu Pharmaceutical Co., Ltd. | *Panax ginseng* C.A.Mey.[Araliaceae]; *Ophiopogon japonicus* (Thunb.) Ker Gawl. [Asparagaceae] | Y - Z51020552 issued by China Food and Drug Administration | N |
| Feng G 2019 | Shenmai injection | Zhengda Qingchunbao Pharmaceutical Co., Ltd. | *Panax ginseng* C.A.Mey.[Araliaceae]; *Ophiopogon japonicus* (Thunb.) Ker Gawl. [Asparagaceae] | Y - Z33020021 issued by China Food and Drug Administration | N |
| Chen TF 2020 | Shenmai injection | Zhengda Qingchunbao Pharmaceutical Co., Ltd. | *Panax ginseng* C.A.Mey.[Araliaceae]; *Ophiopogon japonicus* (Thunb.) Ker Gawl. [Asparagaceae] | Y - Z33020018 issued by China Food and Drug Administration | N |
| Liu LQ 2013 | Shengmai injection | Jiangsu Suzhong Construction Group Co., Ltd. | *Panax ginseng* C.A.Mey.[Araliaceae]; *Ophiopogon japonicus* (Thunb.) Ker Gawl [Asparagaceae]; *Schisandra chinensis* (Turcz.) Baill. [Schisandra chinensis] | Y - Z32021056 issued by China Food and Drug Administration | N |
| Ai HL 2013 | Shenqifuzheng injection | Not mentioned | *Astragalus mongholicus* Bunge [Fabaceae]; *Codonopsis pilosula* (Franch.) Nannf [Campanulaceae]. | N | N |
| Ma JQ 2018 | Shenqifuzheng injection | Not mentioned | *Astragalus mongholicus* Bunge [Fabaceae]; *Codonopsis pilosula* (Franch.) Nannf [Campanulaceae]. | Y - Z19990065 issued by China Food and Drug Administration | N |
| Ren Y 2013 | Huangqi injection | Zhengda Qingchunbao Pharmaceutical Co., Ltd. | *Astragalus mongholicus* Bunge [Fabaceae] | Y - Z330220179 issued by China Food and Drug Administration | N |
| Chen YB 2008 | Huangqi injection | Chengdu Diao Pharmaceutical group Co., Ltd. | *Astragalus mongholicus* Bunge [Fabaceae] | Y - China lot number: 0709009 | N |
| Zhang N 2017 | Shenfu injection | Ya'an Sanjiu Pharmaceutical Co., Ltd. | *Panax ginseng* C.A.Mey.[Araliaceae]; *Aconitum carmichaeli Debeaux* [Ranunculaceae] | Y - Batch Number.: Z20043117 |  |
