# Supplementary material for: Comparative Efficacy of Tonic Chinese Herbal Injections for Treating Sepsis or Septic Shock: A Systematic Review and Bayesian Network Meta-Analysis of Randomized Controlled Trials
Source: Front Pharmacol. 2022 Mar 15;13:830030. doi: 10.3389/fphar.2022.830030 (PMC8972587; doi:10.3389/fphar.2022.830030)
Supplement: Supplementary file 3 [file DataSheet2.docx]

**Attachment 2: Search Strategy**

PubMed

| Number | Search terms | Results |
| --- | --- | --- |
| #1 | Sepsis[MeSH Terms] | 129047 |
| #2 | Septic shock[MeSH Terms] | 23406 |
| #3 | Systemic inflammatory response syndrome[MeSH Terms] | 134640 |
| #4 | Sepsis[Title/Abstract] | 105595 |
| #5 | Septic shock[Title/Abstract] | 24195 |
| #6 | Systemic inflammatory response syndrome[Title/Abstract] | 5363 |
| #7 | SIRS[Title/Abstract] | 5889 |
| #8 | #1 OR #2 OR #3 OR #4 OR #5 OR #6 OR #7 | 206027 |
| #9 | Chinese herbal injection[MeSH Terms] | 1164 |
| #10 | Chinese herbal injection[Title/Abstract] | 33 |
| #11 | Traditional Chinese medicine [MeSH Terms] | 20736 |
| #12 | Traditional Chinese medicine [Title/Abstract] | 23351 |
| #13 | Shenfu[Title/Abstract] | 245 |
| #14 | Shenmai[Title/Abstract] | 245 |
| #15 | Shengmai[Title/Abstract] | 215 |
| #16 | Huangqi[Title/Abstract] | 323 |
| #17 | Shenqifuzheng[Title/Abstract] | 15 |
| #18 | #9 OR #10 OR #11 OR #12 OR #13 OR #14 OR #15 OR #16 OR #17 | 37861 |
| #19 | randomized controlled trial[Publication Type] | 537795 |
| #20 | controlled clinical trial[Publication Type] | 627193 |
| #21 | random*[All Fields] | 1291118 |
| #22 | #20 OR #21 OR #22 | 1375004 |
| #23 | #8 AND #18 AND #22 | 78 |

Web of Science

| Number | Search terms | Results |
| --- | --- | --- |
| #1 | TOPIC: Sepsis | 101535 |
| #2 | TOPIC: Septic shock | 33698 |
| #3 | TOPIC: Systemic inflammatory response syndrome | 8853 |
| #4 | TOPIC: SIRS | 20220 |
| #5 | #1 OR #2 OR #3 OR #4 | 135510 |
| #6 | TOPIC: Chinese herbal injection | 661 |
| #7 | TOPIC: Traditional Chinese medicine | 28501 |
| #8 | TOPIC: Shenfu | 275 |
| #9 | TOPIC: Shenmai | 144 |
| #10 | TOPIC: Shengmai | 155 |
| #11 | TOPIC: Huangqi | 286 |
| #12 | TOPIC: Shenqifuzheng | 13 |
| #13 | #6 OR #7 OR #8 OR #9 OR #10 OR #11 OR #12 | 29491 |
| #14 | #5 AND #13 | 264 |

Cochrane Library

| Number | Search terms | Results |
| --- | --- | --- |
| #1 | Sepsis:ti,ab,kw OR Septic shock:ti,ab,kw OR Systemic inflammatory response syndrome:ti,ab,kw OR SIRS:ti,ab,kw | 15122 |
| #2 | Chinese herbal injection:ti,ab,kw OR Traditional Chinese medicine:ti,ab,kw OR Shenfu:ti,ab,kw OR Shenmai:ti,ab,kw | 8490 |
| #3 | Shengmai:ti,ab,kw OR Huangqi:ti,ab,kw OR Shenqifuzheng:ti,ab,kw | 472 |
| #4 | #2 OR #3 | 8890 |
| #5 | randomized controlled trial:pt OR controlled clinical trial:pt OR random | 1280231 |
| #6 | #1 AND #4 AND #5 | 31 |

Embase

| Number | Search term | Results |
| --- | --- | --- |
| #1 | 'sepsis':ti,ab,kw OR 'septic shock':ti,ab,kw OR 'Systemic inflammatory response syndrome':ti,ab,kw OR 'SIRS':ti,ab,kw OR 'sepsis':exp OR 'septic shock':exp OR 'Systemic inflammatory response syndrome':exp | 349767 |
| #2 | 'Chinese herbal injection':ti,ab,kw OR 'Traditional Chinese medicine':ti,ab,kw OR 'Shenfu':ti,ab,kw OR 'Shenmai':ti,ab,kw OR 'Shengmai':ti,ab,kw OR 'Huangqi':ti,ab,kw OR 'Shenqifuzheng':ti,ab,kw OR 'Chinese medicine':exp OR 'Shenfu':exp OR 'Shengmai':exp | 69782 |
| #3 | 'randomized controlled trial':it OR 'controlled clinical trial':it OR 'random' | 369470 |
| #4 | #1 AND #2 AND #3 | 14 |

CNKI

| Number | Search term | Results |
| --- | --- | --- |
| #1 | (SU='sepsis' OR SU='septic shock' OR SU='severe infection') AND (SU='injection' OR SU='extractive' OR SU='for injection' OR SU='Shenfu' OR SU='Shenmai' OR SU='Shengmai' OR SU='Shenqifuzheng' OR SU='Huangqi') | 2347 |

Wanfang Data

| Number | Search term | Results |
| --- | --- | --- |
| #1 | (SU:'sepsis' OR SU:'septic shock' OR SU:'severe infection') AND (SU:'injection' OR SU:'extractive' OR SU:'for injection' OR SU:'Shenfu' OR SU:'Shenmai' OR SU:'Shengmai' OR SU:'Shenqifuzheng' OR SU:'Huangqi') | 6002 |

VIP

| Number | Search term | Results |
| --- | --- | --- |
| #1 | (ti,kw='sepsis' OR ti,kw='septic shock' OR ti,kw='severe infection') AND (ti,kw='injection' OR ti,kw='extractive' OR ti,kw='for injection' OR ti,kw='Shenfu' OR ti,kw='Shenmai' OR ti,kw='Shengmai' OR ti,kw='Shenqifuzheng' OR ti,kw='Huangqi') | 1300 |

SinoMed

| Number | Search term | Results |
| --- | --- | --- |
| #1 | (ti,kw='sepsis' OR ti,kw='septic shock' OR ti,kw='severe infection' Mesh='sepsis') AND (ti,kw='injection' OR ti,kw='extractive' OR ti,kw='for injection' OR ti,kw='Shenfu' OR ti,kw='Shenmai' OR ti,kw='Shengmai' OR ti,kw='Shenqifuzheng' OR ti,kw='Huangqi') | 2085 |
