# Supplementary material for: Comparative Efficacy of Tonic Chinese Herbal Injections for Treating Sepsis or Septic Shock: A Systematic Review and Bayesian Network Meta-Analysis of Randomized Controlled Trials
Source: Front Pharmacol. 2022 Mar 15;13:830030. doi: 10.3389/fphar.2022.830030 (PMC8972587; doi:10.3389/fphar.2022.830030)
Supplement: Supplementary file 4 [file DataSheet1.docx]

**Table S1**. Node splitting method results of APACHE II score

| Treatment | Direct | | Indirect | | Difference | | P value |
| --- | --- | --- | --- | --- | --- | --- | --- |
|  | Coefficient | Standard Error | Coefficient | Standard Error | Coefficient | Standard Error |  |
| WM vs Shengmai | -1.94 | 1.83 | -1.99 | 3.39 | 0.05 | 3.83 | 0.99 |
| Shenfu vs Shengmai | 0.52 | 1.84 | 0.57 | 3.36 | -0.05 | 3.83 | 0.99 |

**Table S2**. Node splitting method results of 28-day mortality

| Treatment | Direct | | Indirect | | Difference | | P value |
| --- | --- | --- | --- | --- | --- | --- | --- |
|  | Coefficient | Standard Error | Coefficient | Standard Error | Coefficient | Standard Error |  |
| WM vs Shengmai | -0.41 | 0.58 | -0.93 | 1.08 | 0.52 | 1.22 | 0.67 |
| Shenfu vs Shengmai | -0.06 | 0.59 | 0.46 | 1.06 | -0.52 | 1.22 | 0.67 |

**Table S3**. Node splitting method results of PCT

| Treatment | Direct | | Indirect | | Difference | | P value |
| --- | --- | --- | --- | --- | --- | --- | --- |
|  | Coefficient | Standard Error | Coefficient | Standard Error | Coefficient | Standard Error |  |
| WM vs Shenfu | -2.64 | 0.76 | -3.52 | 4.69 | 0.88 | 4.75 | 0.85 |
| WM vs Shengmai | -1.15 | 1.14 | -5.88 | 3.75 | 4.73 | 3.92 | 0.23 |
| Shenfu vs Shengmai | 0.09 | 2.06 | 1.75 | 1.62 | -1.66 | 2.62 | 0.53 |

**Table S4**. Node splitting method results of CRP

| Treatment | Direct | | Indirect | | Difference | | P value |
| --- | --- | --- | --- | --- | --- | --- | --- |
|  | Coefficient | Standard Error | Coefficient | Standard Error | Coefficient | Standard Error |  |
| WM vs Shenfu | -3.63 | 7.45 | -17.64 | 39.96 | 14.01 | 40.70 | 0.73 |
| WM vs Shengmai | 1.39 | 9.50 | -16.21 | 36.03 | 17.60 | 37.21 | 0.64 |
| Shenfu vs Shengmai | 2.92 | 18.98 | 5.14 | 13.61 | -2.22 | 23.32 | 0.53 |
